# A non-catalytic interaction surface mediates recognition of spliceosomal factors by the *Toxoplasma gondii* cyclophilin TgCyp23

**DOI:** 10.1042/BCJ20260439

**Published:** 2026-09-03

**Authors:** Filippo Favretto, Silvia Fruncillo, Eva Jiménez-Faraco, Federico Gago, Paola Dominici, Juan A. Hermoso, Alessandra Astegno

**Affiliations:** 1 Department of Biotechnology, University of Verona, Verona, Italy; 2 Department of Crystallography and Structural Biology, Institute of Physical Chemistry Blas Cabrera (IQF), CSIC, Madrid, Spain; 3 Department of Biomedical Sciences, School of Medicine and Health Sciences, University of Alcalá, Alcalá de Henares, Spain

**Keywords:** crystal structure, Cyclophilins, cyclosporin A, protein-peptide interactions, spliceosome, Toxoplasma gondii

## Abstract

The spliceosome is emerging as a key regulatory hub in *Toxoplasma gondii*, yet the molecular basis of spliceosomal protein interactions remains largely unexplored. TgCyp23, a predicted nuclear cyclophilin (Cyp) from *T. gondii*, shares sequence similarity and catalytic properties with the human spliceosomal cyclophilin H (hCypH) that interacts with the splicing factors PRP4 and PRP18. Here, we investigated whether TgCyp23 engages in analogous interactions with the *T. gondii* orthologs TgPRP4 and TgPRP18 using peptides corresponding to their predicted Cyp-binding regions. High-resolution crystal structures of TgCyp23 in complex with a TgPRP4-derived peptide, including a ternary complex with the Cyp inhibitor cyclosporin A, reveal that spliceosomal partner recognition occurs outside the catalytic site, which remains fully accessible. NMR analyses extend this binding mode to TgPRP18, demonstrating that peptides derived from both spliceosomal factors engage the same surface in solution. Isothermal titration calorimetry shows that TgCyp23 binds both peptides with low- to mid-micromolar affinity, while circular dichroism and molecular dynamics support a folding-upon-binding mechanism. Notably, binding of either peptide does not affect the peptidyl-prolyl isomerase activity of TgCyp23 or its sensitivity to cyclosporin A, indicating that partner recognition and catalytic function are mechanistically separable. Together, these findings identify a non-catalytic interaction surface in TgCyp23 that mediates recognition of spliceosomal factors. The similar interaction mode observed for TgCyp23 and hCypH suggests that spliceosomal partner recognition may be conserved and supports a role for TgCyp23 as a spliceosome-associated Cyp in *T. gondii*, providing a structural framework for understanding Cyp interactions within the parasite spliceosome.

## Introduction

The spliceosome is a central regulator of gene expression in eukaryotes and a highly dynamic macromolecular machine responsible for accurate pre-mRNA splicing. By orchestrating the precise removal of introns and ligation of exons, the spliceosome plays a fundamental role in controlling transcript diversity, developmental programs, and cellular adaptation.

At the molecular level, the spliceosome is composed of U-rich small nuclear RNAs (snRNAs) that assemble with numerous protein partners into small nuclear ribonucleoproteins (snRNPs). These snRNPs dynamically associate with regulatory proteins at different stages of the splicing cycle, enabling accurate splice-site recognition and efficient catalysis [1].

Among the proteins that associate with the spliceosome are cyclophilins (Cyps), a ubiquitous and evolutionarily conserved family of peptidyl-prolyl *cis–trans* isomerases (PPIases) involved in protein folding, signaling, and stress responses [2,3]. In addition to their catalytic PPIase domain, Cyps often contain auxiliary structural elements that mediate diverse protein–protein interactions. When incorporated into the spliceosome, these proteins are collectively referred to as spliceophilins [4].

In humans, eight spliceophilins have been identified, hCypE, hCypG, hCypH, hPPIL1, hPPIL2, hPPIL3, hPPWD1, and hCWC27, each fulfilling distinct and non-redundant roles in splicing regulation. Their diverse domain architectures enable stage-specific integration into spliceosomal complexes, where many of them contribute to protein–protein interactions in addition to their potential peptidyl-prolyl isomerase activity [5,6]. Among human spliceophilins, hCypH is a key component of the splicing machinery [1,5,7–9]. hCypH is present in the stand-alone tri-snRNP and is incorporated into the spliceosome during formation of the B complex but dissociates during spliceosome activation, prior to the pre-catalytic B_act_ stage [1]. Functional studies have shown that the immunosuppressive Cyp inhibitor cyclosporin A (CsA) slows splicing steps involving hCypH, while expression of a CsA-resistant hCypH variant prevents CsA-mediated splicing inhibition, demonstrating the functional importance of hCypH in splicing [5]. Mechanistically, hCypH interacts with the splicing factors hPRP4 and hPRP18, which participate in spliceosome assembly before the first catalytic step and during the second catalytic step, respectively. Importantly, these interactions occur outside the hCypH active site and are not disrupted by CsA binding [5,7,10].

Spliceosomal regulation has recently gained increasing attention as a potential target for antimicrobial therapies. Perturbation of spliceosome integrity in protozoan parasites [11], including *Trypanosoma brucei* and *Toxoplasma gondii*, results in reduced virulence, impaired development, and severe fitness defects, underscoring the therapeutic potential of targeting splicing-related processes [12,13]. Notably, more than 75% of *T. gondii* genes contain introns, compared with approximately 5% in yeast [14,15]. This exceptionally high intron density suggests that intron recognition and alternative splicing have been key evolutionary features in *T. gondii*, enabling fine-tuning of gene expression across multiple developmental stages and adaptation to diverse host environments. Consistent with this complexity, the *T. gondii* genome encodes a complete and elaborate spliceosomal machinery. As in humans, the *T. gondii* genome encodes multiple putative Cyps (TgCyps) [3]. However, only a limited subset of TgCyps have been characterized to date [16–21], while the majority remain unexplored. Among the characterized members, TgCyp21 and TgCyp23 have been shown to possess PPIase activity and to be sensitive to CsA inhibition [20,22,23]. Notably, both TgCyp21 and TgCyp23 are predicted to localize to the nucleus, raising the possibility that they may participate in spliceosomal-associated processes in the parasite.

In this work, we investigated whether TgCyp23 behaves as a spliceophilin *in vitro* by engaging predicted binding regions from the *T. gondii* orthologs of the human splicing factors PRP4 and PRP18. Establishing these interactions is essential to determine whether, beyond its peptidyl-prolyl isomerase activity, TgCyp23 contributes to the molecular organization of the parasite spliceosome by providing a platform for spliceosomal protein–protein interactions. Based on sequence conservation with hCypH, we identified the putative TgPRP4 and TgPRP18 orthologs and designed peptides corresponding to their predicted TgCyp23-binding regions (TgPRP4p and TgPRP18p). Using a combination of structural, biophysical and enzymatic approaches, we demonstrate that TgCyp23 forms stable complexes with both peptides while retaining its catalytic activity and sensitivity to CsA. We determined the crystal structures of the TgCyp23–TgPRP4p complex at 2.4 Å resolution and of the ternary TgCyp23–TgPRP4p–CsA complex at 1.25 Å resolution, revealing that peptide binding occurs on a surface distinct from the catalytic site. Complementary nuclear magnetic resonance (NMR) analyses demonstrated that TgPRP18p binds the same surface in solution. Together, these findings identify a conserved protein–protein interaction interface in TgCyp23 that mediates recognition of spliceosomal partners while leaving the catalytic site accessible.

## Results

### Sequence analysis identifies conserved hCypH interaction determinants in TgCyp23 and its putative spliceosomal partners

TgCyp23 is predicted to localize to the nucleus according to the *Toxoplasma* genome database (https://toxodb.org/toxo/). A search of the NCBI database using TgCyp23 as the query identified hCypH as its closest human homolog, with an *E*-value of 4 × 10^−83^ and >60% sequence identity, indicating a high degree of evolutionary conservation.

**Figure 1:**
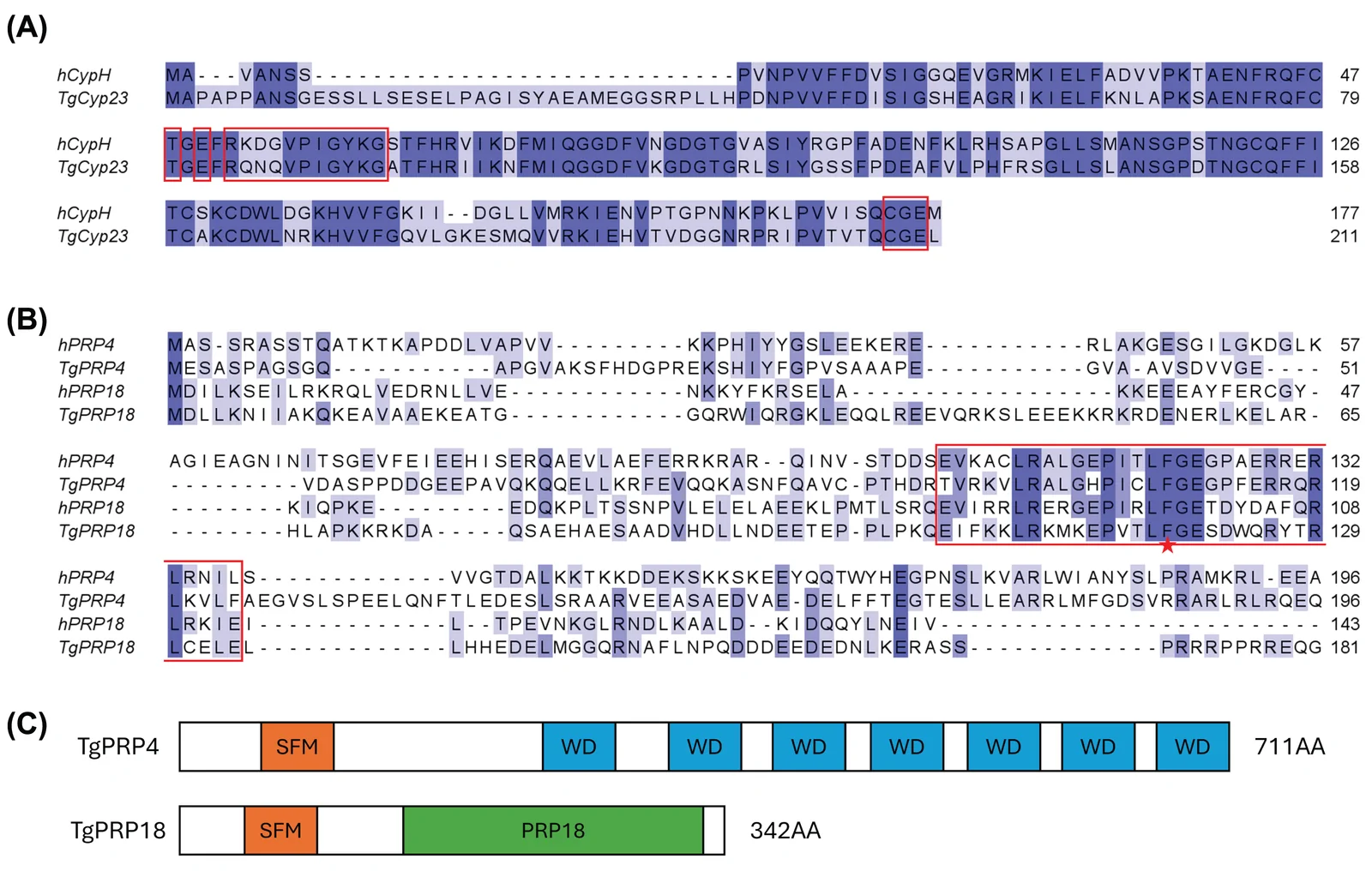
Sequence and domain analysis of TgCyp23 and its putative spliceosomal partners (**A**) Sequence alignment of hCypH (UniProt ID: O43447) and TgCyp23 (UniProt ID: A0A125YL73). (**B**) Sequence alignment of the N-terminal regions of human PRP4 (hPRP4; UniProt ID: O43172), *T. gondii* PRP4 (TgPRP4; UniProt ID: S8F6Y9), human PRP18 (hPRP18; UniProt ID: Q99633), and *T. gondii* PRP18 (TgPRP18; UniProt ID: A0A125YI37). In both alignments (panels A and B), residues or regions implicated in Cyp binding are highlighted in red boxes, whereas residues known to be critical for binding are marked with a star. (**C**) Schematic representation of the domain organization of TgPRP4 and TgPRP18, showing the N-terminal SFM which mediates Cyp binding.

Sequence alignment between TgCyp23 and hCypH revealed that all residues previously shown to mediate interactions between hCypH and the splicing factors hPRP4 and hPRP18 are conserved in TgCyp23 (Figure 1A). In particular, Thr48 and Glu50 of hCypH (corresponding to Thr80 and Glu82 in TgCyp23), which interact with Lys109 and Gly123 of hPRP4, are preserved [7]. Moreover, the loop spanning residues Arg52–Lys61, a structural element characteristic of hCypH and absent in hCypA, which contributes to the formation of a sandwich-like interaction around the Phe122 of hPRP4, is also conserved in TgCyp23. Consistently, the C-terminal segment of hCypH (Cys174–Glu176), which contacts Arg113, Ile119, Thr120, and Phe122 of hPRP4, is likewise conserved in TgCyp23 [5,7].

The *T. gondii* genome also encodes orthologs of the splicing factors hPRP4 and hPRP18, namely TgPRP4 (Uniprot ID: S8F6Y9) and TgPRP18 (Uniprot ID: A0A125YI37). Both hPRP4 and hPRP18 contain a conserved 31-residue Cyp-binding region within their N-terminal splicing factor motif (SFM), which mediates direct association with hCypH. This region is well conserved in TgPRP4 and TgPRP18 (Figure 1B,C). In particular, the key phenylalanine residue (Phe122 in hPRP4), which inserts into the hydrophobic cleft of hCypH, is retained together with neighboring residues Gly123, Arg113, Val108, Leu112, Arg132, Leu133, and the structurally important Pro118 [5,7].

### TgCyp23 recognizes the predicted cyclophilin-binding motifs of TgPRP4 and TgPRP18

To assess whether TgCyp23 interacts with its predicted spliceosome partners, we synthesized 31-residue peptides corresponding to the putative Cyp-binding regions of TgPRP4 (TgPRP4p, residues 94–124: TVRKVLRALGHPICLFGEGPFERRQRLKVLF) and TgPRP18 (TgPRP18p, residues 104–134: EIFKKLRKMKEPVTLFGESDWQRYTRLCELE), identified by sequence comparison with their human orthologs. These peptides were then used for a comprehensive biophysical and structural characterization of their interaction with recombinant TgCyp23.

**Figure 2:**
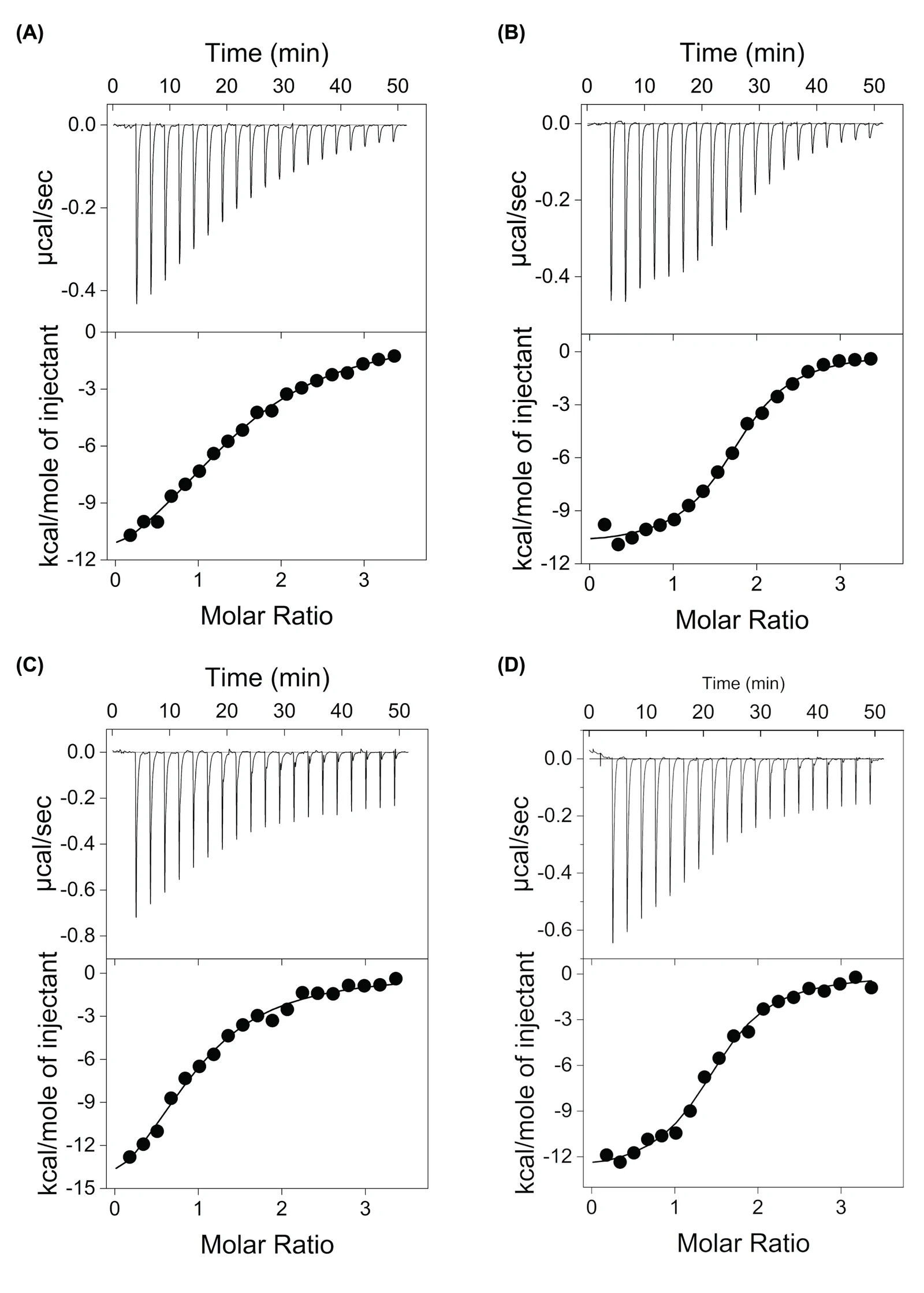
ITC analysis of peptide binding to TgCyp23 Representative ITC thermograms (upper panels) and integrated binding isotherms (lower panels) recorded at 25°C for TgCyp23 titrated with (**A**) TgPRP18p and (**B**) TgPRP4p. (**C,D**) ITC experiments were performed with TgCyp23 pre-incubated with CsA and subsequently titrated with (**C**) TgPRP18p and (**D**) TgPRP4p. All measurements were carried out in 50 mM Hepes, pH 8.0, and 100 mM NaCl.

Peptide binding was first examined by ITC. Representative ITC thermograms are shown in Figure 2, and the derived thermodynamic parameters are summarized in Table 1. ITC measurements showed that both TgPRP4p and TgPRP18p bind TgCyp23 through a single exothermic binding event (Figure 2A,B). TgPRP4p bound TgCyp23 with a dissociation constant (*K*_d_) of 3.3 ± 0.3 μM, comparable to the affinity reported for the interaction between hCypH and the hPRP4 binding region (∼2 μM) [7]. In contrast, TgPRP18p displayed a lower affinity, with a *K*_d_ of 18 ± 5 μM, corresponding to an approximately 5-fold weaker interaction.

**Table 1:** Thermodynamic parameters of the interaction of TgCyp23 with TgPRP4p or TgPRP18p

Protein	Peptide	*N*	*K*_d_ (μM)	Δ*H* (kcal/mol)	–TΔ*S* (kcal/mol)
TgCyp23	TgPRP18p	1.2 ± 0.2	18 ± 5	–17.9 ± 2.9	10.8 ± 2.8
TgCyp23	TgPRP4p	1.5 ± 0.2	3.3 ± 0.3	–15.7 ± 1.5	8.2 ± 1.5
TgCyp23 + CsA	TgPRP18p	1.1 ± 0.1	13.3 ± 4.8	–15.4 ± 2.1	8.7 ± 1.9
TgCyp23 + CsA	TgPRP4p	1.4 ± 0.2	4.6 ± 0.3	–11.8 ± 2.5	7.2 ± 1.1

To rationalize this difference in binding affinity, molecular dynamics (MD) simulations were performed on both complexes. After 500 ns of simulation in aqueous solution, both complexes remained structurally stable. In each case, peptide binding was maintained through a persistent interaction interface that included the formation of a short antiparallel β-sheet involving TgCyp23 residues Cys208 and Glu210 (Supplementary Table S1). Key TgCyp23 residues involved in peptide binding are summarized in Supplementary Figure S1A. Both complexes were primarily stabilized by van der Waals and electrostatic contacts, which together account for most of the interaction energy (Supplementary Figure S1B). Additional contributions arose from peptide and protein desolvation, as well as apolar interactions, that further modulate the overall binding energetics, although to a lesser extent. In both complexes, electrostatic contributions arose from hydrogen bonds involving conserved residues. Specifically, Phe109 in TgPRP4 and Phe119 in TgPRP18, along with residues Ile106 and Cys107 in TgPRP4 or Val116 and Thr117 in TgPRP18, established hydrogen bonds with Cys208 and Glu210 of TgCyp23. The hydrogen bond distances remained stable throughout the simulation in both complexes (Supplementary Figure S1C and S1D). Notably, in the TgCyp23–TgPRP4p complex, the N-terminal Arg96 and Arg100 of the peptide reoriented during the simulation to establish favorable ionic interactions with Asp41, Glu210 and the C-terminal carboxylate of TgCyp23, thereby contributing additional binding energy. In contrast, the equivalent position in TgPRP18p is occupied by a phenylalanine residue (Phe106), which results in weakened electrostatic interactions. Consistently, the α-helix of TgPRP4p underwent a slight reorientation that further stabilized the interaction between Arg96 and the TgCyp23 C-terminus. Together, these observations provide a structural and dynamic rationale for the higher binding affinity of TgPRP4p compared to TgPRP18p.

ITC experiments further showed that preincubation of TgCyp23 with CsA did not affect binding to either TgPRP4p or TgPRP18p (Figure 2C,D and Table 1), suggesting that peptide binding occurs outside the CsA-sensitive catalytic site. Moreover, the specificity of these interactions was confirmed by control ITC experiments showing that two other *T. gondii* Cyps available in our laboratory, TgCyp21 and TgCyp18, do not bind either peptide under identical conditions (Supplementary Figure S2).

**Figure 3:**
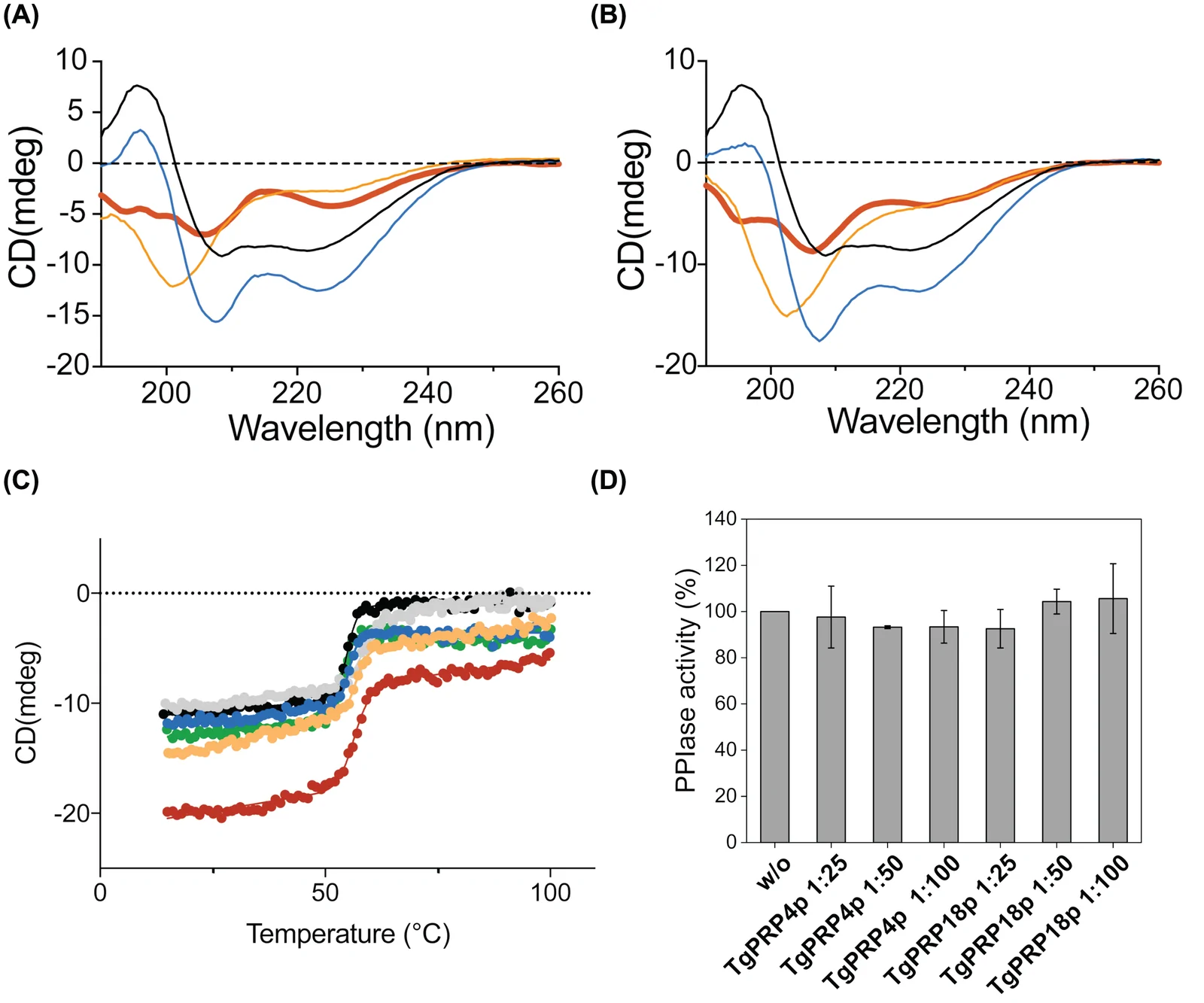
Biophysical characterization of TgCyp23 upon peptide binding (**A,B**) Far-UV CD spectra of TgCyp23 in complex with (**A**) TgPRP18p and (**B**) TgPRP4p. The spectra correspond to 0.2 mg ml^−1^ TgCyp23 (black), 0.5 mg ml^−1^ TgPRP4p or TgPRP18p (orange), the protein–peptide complex at 1:5 molar ratio (blue), and the difference spectrum (red), obtained by subtracting the spectrum of TgCyp23 alone from that of the corresponding protein–peptide complex. (**C**) Thermal denaturation profiles of TgCyp23 alone (black), TgCyp23–TgPRP4p (blue; protein:peptide molar ratio: 1:5), TgCyp23–TgPRP18p (green; protein:peptide molar ratio: 1:5), TgCyp23–CsA (gray; protein:CsA molar ratio: 1:2), TgCyp23–CsA–TgPRP4p (red; protein:CsA:peptide molar ratio: 1:2:5), and TgCyp23–CsA–TgPRP18p (orange; protein:CsA:peptide molar ratio: 1:2:5). (**D**) PPIase activity of TgCyp23 measured in the presence of increasing molar excess (25-, 50-, or 100-fold) of TgPRP4p or TgPRP18p. Activity was determined using the chymotrypsin-coupled assay with AAPF as substrate and is reported relative to untreated TgCyp23 (set to 100%).

Thermodynamic analysis revealed that the interactions of TgCyp23 with both TgPRP4p and TgPRP18p were characterized by a favorable enthalpic contribution (Δ*H* <0) and an unfavorable entropic term (Δ*S* <0), suggesting increased structural order upon complex formation. To further investigate this aspect, far-UV circular dichroism (CD) spectroscopy was employed. The isolated peptides displayed spectra typical of intrinsically disordered polypeptides, with a minimum around 205 nm, whereas TgCyp23 exhibited pronounced minima at 208 and 222 nm, indicative of a predominantly α-helical fold, in agreement with previous reports (Figure 3A,B) [20]. Upon peptide binding, the CD spectra of the complexes showed an increase in ellipticity at 222 nm accompanied by a decrease at 205 nm. Difference CD spectra, obtained by subtracting the spectrum of TgCyp23 alone from that of the corresponding TgCyp23–peptide complex, isolated the CD contribution of the peptide in the bound state. These spectra displayed the characteristic minima at 208 and 222 nm, indicating that the peptides acquired α-helical secondary structure upon binding to TgCyp23. The extent of this disorder-to-order transition increased with peptide concentration and was most pronounced at a 1:5 protein-to-peptide molar ratio. Moreover, consistent with an intrinsic folding propensity, both peptides adopted α-helical character in the presence of increasing concentrations of TFE, supporting the notion that binding to TgCyp23 stabilizes a pre-existing helical tendency (Supplementary Figure S3). This folding-upon-binding mechanism provides a structural explanation for the unfavorable entropic contribution observed by ITC and mirrors the behavior previously described for the interaction of hPRP4 with hCypH [7]. MD simulations demonstrated that the conformations of the modeled fragments of both TgPRP4p and TgPRP18p in solution were notably stable, mostly due to hydrophobic collapse (Supplementary Figs S4 and S5). Consistent with this behavior, the backbone φ and ψ torsion angles, which describe rotations around the N-Cα and Cα-C bonds, displayed well-defined average values with relatively small standard deviations, indicating limited backbone flexibility.

Thermal denaturation experiments monitored by CD at 224 nm showed that the apparent melting temperature (*T_m,app_*) of TgCyp23 remained essentially unchanged upon binding of either TgPRP4p or TgPRP18p, both in the absence and presence of CsA (Figure 3C and Supplementary Table S2). In line with our previous findings [20], pre-incubation with CsA increased the *T_m,app_* of TgCyp23 by approximately 3°C. The *T_m,app_* values of the TgCyp23–CsA–TgPRP4p and TgCyp23–CsA–TgPRP18p complexes were comparable to the value for the TgCyp23−CsA complex, indicating that peptide binding does not substantially alter the stabilizing effect of CsA (Supplementary Table S2). Importantly, TgCyp23 catalytic activity remained unchanged in the presence of increasing concentrations of TgPRP4p or TgPRP18p, even at a 100-fold molar excess (Figure 3D). TgCyp23 also remained fully sensitive to CsA inhibition when complexed with a 50-fold molar excess of either peptide, with an IC_50_ of ∼1 nM, in line with previous measurements (Supplementary Table S3 and Supplementary Figure S6) [20]. Together, these results demonstrate that TgPRP4p and TgPRP18p bind TgCyp23 through a non-catalytic interaction surface, leaving the active site accessible and functionally competent.

### Crystal structures of TgCyp23–TgPRP4p and TgCyp23–TgPRP4p–CsA complexes

The crystal structure of TgCyp23–CsA (PDB: 8B58) was previously reported [20], revealing a conserved fold shared with other Cyps, comprising eight antiparallel β-strands sandwiched between two long α-helices [3,22]. Here, we solved two new structures: the TgCyp23–TgPRP4p complex and the TgCyp23–TgPRP4p–CsA ternary complex at 2.40 and 1.25 Å resolution, respectively. These structures were obtained by co-crystallization with TgPRP4p and CsA ligands and solved by the molecular replacement method, using a TgCyp23 monomer as the search model. In the TgCyp23–TgPRP4p complex, the asymmetric unit contains one complex molecule, whereas in the TgCyp23–TgPRP4p–CsA complex two copies of the ternary complex were found. In both cases, the omit map clearly showed the presence of TgPRP4p, which was easily placed by comparison with the AlphaFold model for this complex. The high quality of the electron density maps also allowed the unambiguous placement of the CsA ligand. All the statistics and electron density maps (2F_o_-F_c_) for the ligands are reported in Supplementary Table S4 and Supplementary Figure S7.

**Figure 4:**
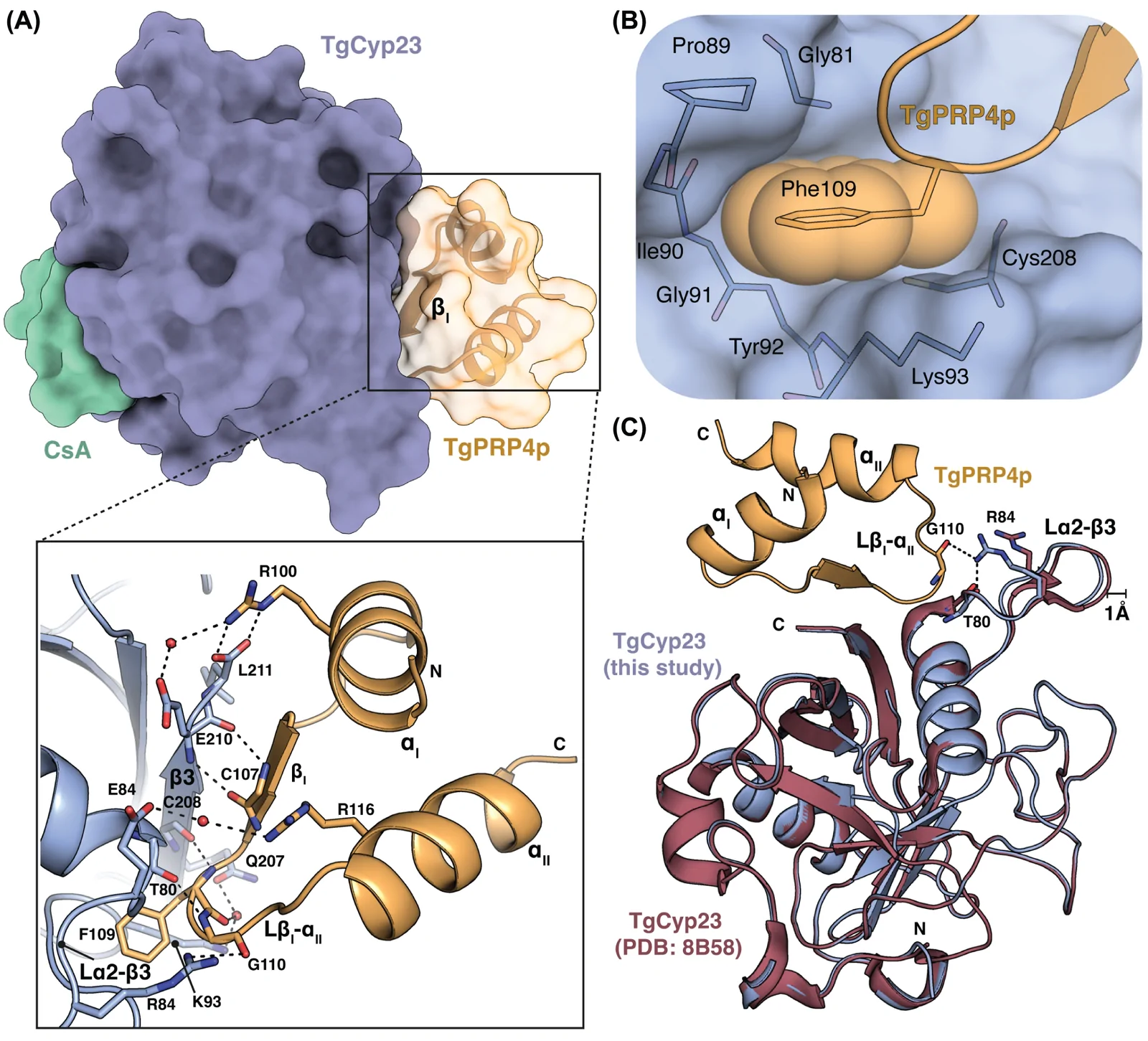
Structural characterization of TgCyp23–TgPRP4p–CsA complex TgCyp23, TgPRP4p, and CsA are colored blue, orange and green, respectively. (**A**) The ternary structure is shown as a surface representation, while TgPRP4p is displayed as a cartoon with a semi-transparent surface. A zoomed-in view is shown at the bottom of the panel, highlighting the main polar interactions between TgCyp23 and TgPRP4p, represented as dashed lines. (**B**) Hydrophobic cavity of TgCyp23 accommodating Phe109 of TgPRP4p. The TgCyp23 pocket is shown as a surface, while the side chain of Phe109 (TgPRP4p) is depicted as semi-transparent spheres. (**C**) Superposition of the TgCyp23–TgPRP4p–CsA complex onto TgCyp23–CsA (PDB: 8B58). The structures are shown in cartoon representation and colored blue and red, respectively. CsA is omitted in both structures, as it is not relevant in this analysis. The label 1 Å indicates the displacement in the Lα2–β3 loop between the two structures. Residues are labeled using one- or three-letter codes. α and β denote α-helices and β-strands, respectively, while L indicates loop regions. N and C denote the N- and C-terminus, respectively.

The ternary TgCyp23–TgPRP4p–CsA structure shows that TgPRP4p and CsA occupy distinct sites, demonstrating that TgPRP4p binding does not preclude inhibitor binding. The CsA binding mode and its interaction pattern with TgCyp23 were extensively described in our previous work [20] and are preserved in the TgCyp23–TgPRP4p–CsA structure. As shown in Figure 4A, TgPRP4p binding does not interfere with the substrate-binding site, as it binds to the opposite face of the catalytic site, where the CsA inhibitor is located. The presence of CsA does not induce any significant conformational change in TgCyp23 as demonstrated by the structural alignment of the binary TgCyp23–TgPRP4p complex and the ternary TgCyp23–TgPRP4p–CsA complex, which show a total RMSD (Root-Mean-Squared-Deviation) between 0.217 and 0.286 Å (for chain D or E of the ternary structure, respectively) (Supplementary Figure S8).

Due to the high resolution of the ternary complex (1.25 Å) and its close structural similarity to the binary complex, we selected TgCyp23–TgPRP4p–CsA as the reference structure to illustrate and discuss the structural details.

TgPRP4p adopts a well-defined conformation within the complex, consisting of two α-helices, αI (residues Val95–Leu102) and αII (Pro113–Leu123), separated by an extended loop and a short β-strand, βI (Ile106–Leu108), that aligns in an antiparallel fashion with β3 of TgCyp23 (Figure 4A, zoomed view). It is worth mentioning that these structural elements in TgPRP4p are preserved in the full-length TgPRP4 as predicted by AF (Supplementary Figure S9). This structural organization promotes the formation of backbone-backbone hydrogen bonds between TgPRP4p residues Cys107, Phe109, Gly110, and TgCyp23 residues Thr80, Cys208, and Glu210. Furthermore, Arg100 and Arg116 (from TgPRP4p αI and αII helices) also interact with TgCyp23 through water-mediated polar interactions with residues Glu210 and Glu84, respectively. In addition, Arg100 of TgPRP4p forms a strong bidentate polar interaction with the carboxylate group of the C-terminal residue Leu211. Moreover, the extended loop of the TgPRP4p peptide (LβI–αII) establishes polar contacts with several residues of the loop Lα2–β3 of TgCyp23. Thr80 and Arg84 from TgCyp23 form hydrogen bonds with the backbone of Gly110 of TgPRP4p, thereby stabilizing the loop conformation and helping to properly orient the bulky side chain of Phe109. Notably, Phe109 of TgPRP4p plays a critical role in protein recognition, as it is buried within a hydrophobic cleft formed by TgCyp23 residues Pro89, Ile90, Gly91, Tyr92, and Lys93. Its phenyl ring is stabilized by van der Waals and hydrophobic interactions (Figure 4B). On one side, it is packed against the proline ring of Pro89 and the α-carbon of Gly91, whereas on the opposite face it rests on the aliphatic chain of Lys93 and the thiol group of Cys208 (forming an SH–π interaction).

To investigate whether there is a conformational change or rearrangement in TgCyp23 upon TgPRP4p binding, we superimposed the TgCyp23–CsA structure (PDB: 8B58) onto the TgCyp23–TgPRP4p–CsA structure (Figure 4C). The superposition showed an RMSD of 0.164 Å, indicating that the protein does not undergo a significant rearrangement upon binding the TgPRP4 peptide. However, a subtle shift was observed at the peptide-binding interface (loop Lα2–β3 of TgCyp23), which is displaced by approximately 1 Å. In this loop, Arg84 changes its conformation and reorients its side chain toward Gly110, forming a hydrogen bond with TgPRP4p.

### Solution NMR reveals a conserved spliceophilin interaction surface on TgCyp23

To gain further insights into the interaction of TgCyp23 with its spliceosomal partners in solution, and to characterize the TgCyp23–TgPRP18p interaction, for which no crystallographic structure is available, we employed NMR spectroscopy. Two-dimensional (2D) ^1^H–^15^N heteronuclear single quantum coherence (^1^H–^15^N HSQC) spectra were acquired using uniformly ^15^N-labeled TgCyp23. Backbone resonance assignments, covering >90% of the protein residues, were previously obtained [23]. Uniformly ^1^H–^15^N-labeled TgCyp23 was titrated with increasing amounts of unlabeled TgPRP18p or TgPRP4p, up to 3-fold molar excess. ^1^H–^15^N HSQC spectra were recorded at each titration point to monitor chemical shift perturbations (CSPs) and signal intensity changes associated with peptide binding.

**Figure 5:**
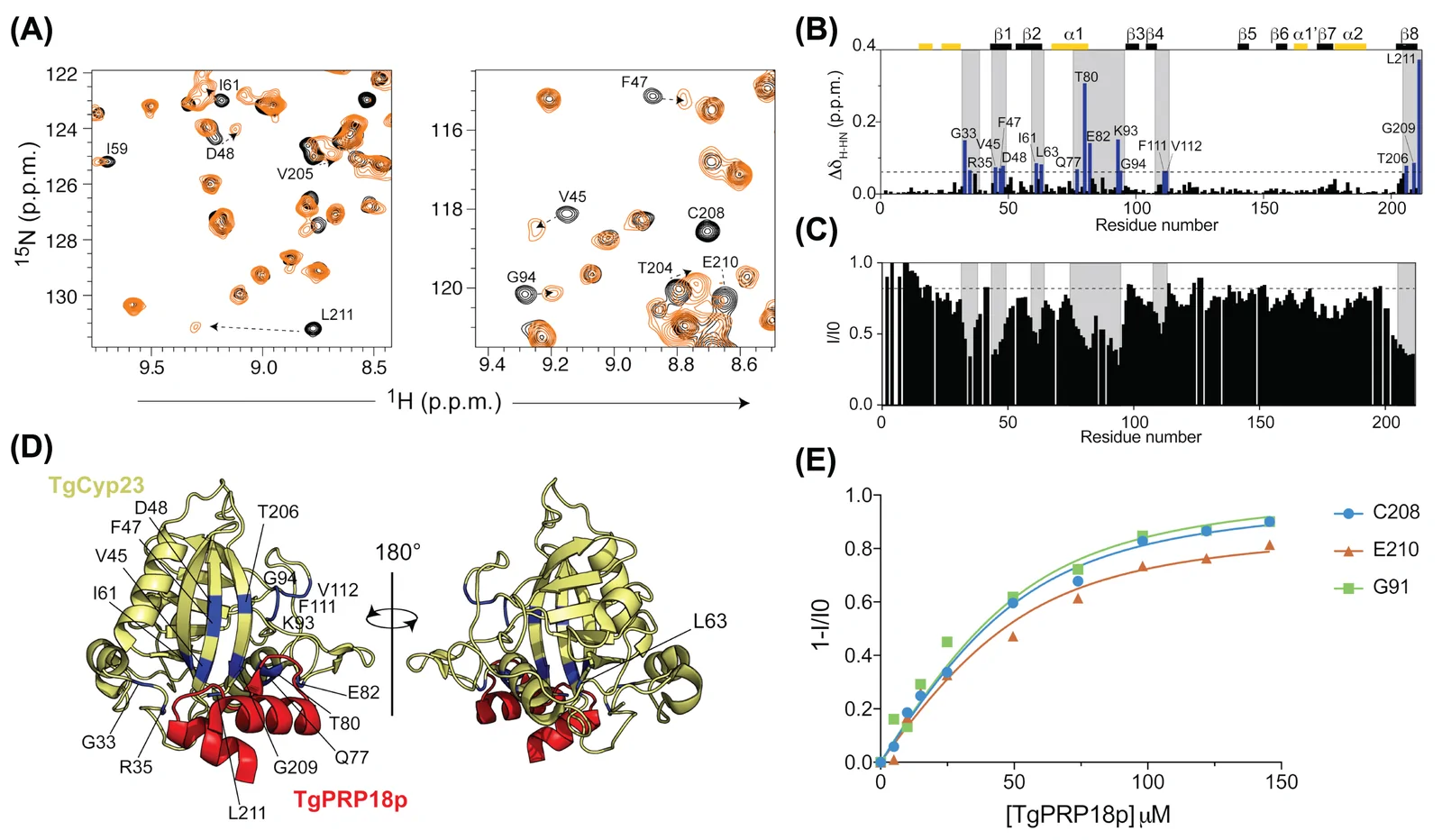
NMR spectroscopy analysis of the interaction between ^15^N–TgCyp23 and TgPRP18p (**A**) Superposition of selected regions of ^15^N–TgCyp23 ^1^H–^15^N HSQC spectrum upon binding of TgPRP18p at a 3-fold molar excess. (**B**) Residue-specific CSPs observed upon interaction of ^15^N–TgCyp23 with TgPRP18p at a molar ratio of 1:3. Secondary structure elements (α-helices in yellow and β-strands in black) are shown above the plot. The dotted line indicates the significance threshold (0.06 ppm), calculated as the average CSP plus one standard deviation. Significantly perturbed residues are labeled. (**C**) Residue-specific attenuation of cross-peak intensities in the ^1^H–^15^N HSQC spectrum of ^15^N–TgCyp23 upon binding of 1.5-fold molar excess of TgPRP18p. (**D**) Mapping of CSPs induced by TgPRP18p onto the three-dimensional structure of TgCyp23 (perturbed residues shown in blue). The model of TgCyp23 (yellow) bound to TgPRP18p (red) was generated by superposition of TgCyp23 (PDB: 8B58) with the hCypH–hPRP4 complex (1MZW) using PyMOL. For illustrative purposes only, TgPRP18p is assumed to adopt a conformation similar to that of hPRP4. (**E**) Representative residue-specific intensity attenuation curves recorded during peptide titration using 50 μM ^15^N–TgCyp23. Curves were globally fitted using a reversible 1:1 binding model to estimate the binding affinity from the observed intensity attenuation.

Upon titration of TgPRP18p, resonances corresponding to the free TgCyp23 gradually decreased in intensity and broadened as the ligand concentration increased, reappearing at different chemical shifts at a 1:3 TgCyp23:TgPRP18p molar ratio, consistent with a slow-to-intermediate exchange regime on the NMR timescale (Figure 5 and Supplementary Figure S10A). Notably, binding of TgPRP18p induced significant CSPs (>0.06 p.p.m.) in several residues, mainly located in β-strands β1, β2 and β8 (C-terminal region), as well as in the α1–β3 and β4–β5 loop regions. Residues exhibiting the largest CSPs also displayed pronounced signal attenuation (Figure 5A–D), consistent with direct involvement in complex formation. Residue-specific intensity attenuation was then analyzed as a function of TgPRP18p concentration and globally fitted using a reversible 1:1 binding model, yielding an estimated *K*_d_ of 15 ± 4 μM, in close agreement with the corresponding ITC-derived value (Figure 5E).

Mapping the CSPs and intensity losses onto the TgCyp23 structure revealed an interaction surface that largely overlaps with that previously described for hCypH in complex with hPRP4 and hPRP18 [7,10]. In particular, Thr80 and Glu82 displayed marked chemical shift changes, as well as intensity loss consistent with their established role in spliceosomal partner recognition. Additional perturbations were observed for Lys93 and Gly94, within the α1–β3 loop, a region known to differ in length among Cyps and to contribute to ligand specificity. At the C-terminus, residues such as Cys208 and Glu210 were strongly affected, in line with their involvement in binding in the human ortholog [5,7]. Perturbations detected in the α1 helix and the β1–β2 region may additionally reflect local conformational rearrangements of TgCyp23 upon TgPRP18p binding.

**Figure 6:**
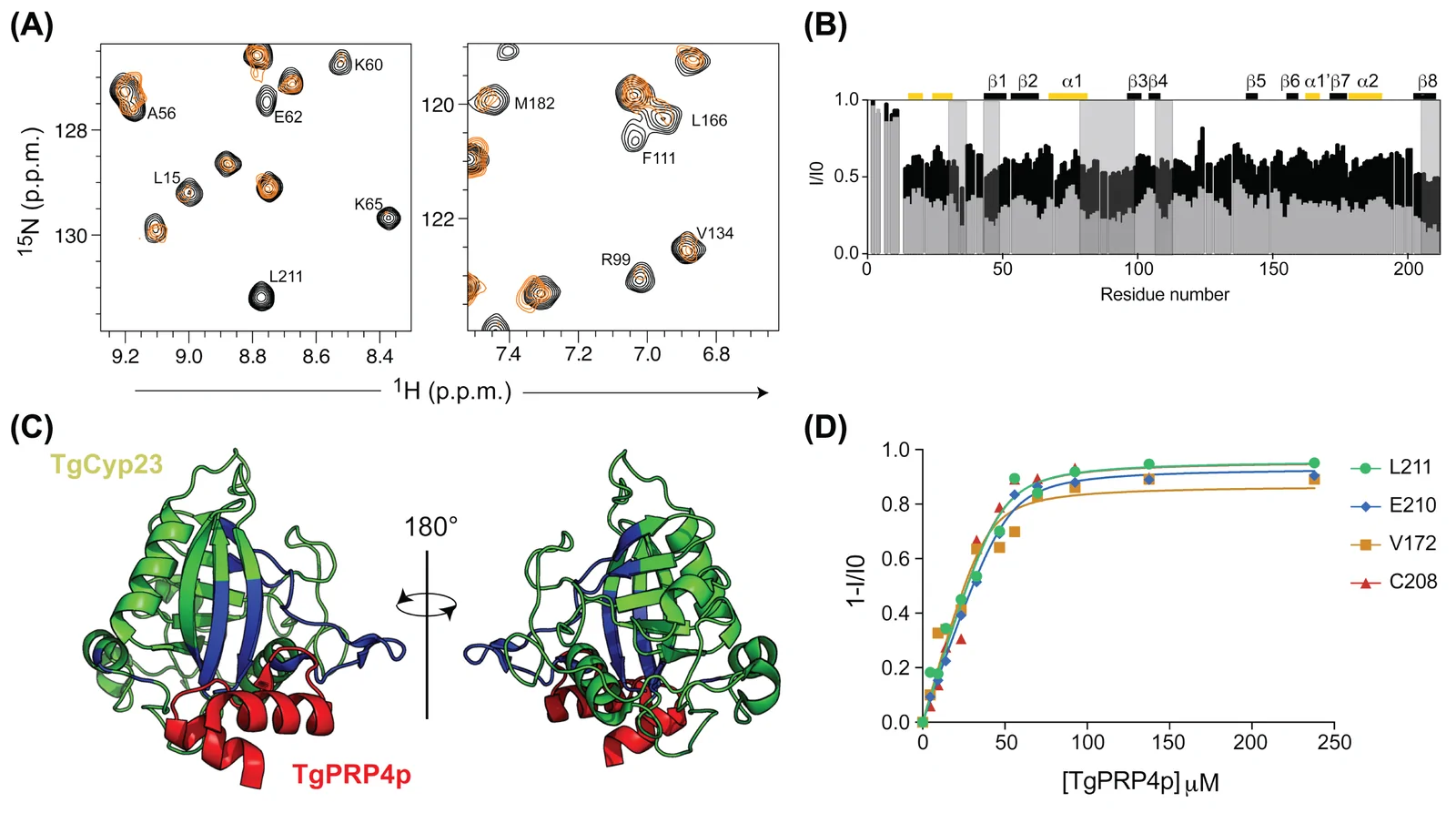
NMR spectroscopy analysis of the interaction between ^15^N–TgCyp23 and TgPRP4p (**A**) Superposition of selected regions of ^15^N–TgCyp23 ^1^H–^15^N HSQC spectrum upon binding of TgPRP4p at a 3-fold molar excess. (**B**) Residue-specific attenuation of cross-peaks intensity of ^1^H–^15^N HSQC spectrum of ^15^N–TgCyp23 upon binding of TgPRP4p at 0.5-fold (black) and 1.5-fold (gray) molar excess of TgPRP4p. Secondary structural elements (α-helices in yellow and β-strands in black) are displayed above the plot. (**C**) Mapping of residue-specific intensity attenuation induced by TgPRP4p onto the three-dimensional structure of TgCyp23 (affected residues shown in blue). The model of TgCyp23 (green) bound to TgPRP4p (red) was obtained by superposition of TgCyp23 (PDB: 8B58) with the hCypH–hPRP4 complex (PDB ID: 1MZW) using PyMOL. (**D**) Representative residue-specific intensity attenuation curves recorded during peptide titration using 50 μM ^15^N–TgCyp23. Curves were globally fitted using a reversible 1:1 binding model to estimate the binding affinity from the observed intensity attenuation.

In contrast, titration of TgCyp23 with TgPRP4p produced a distinct spectral response, characterized by extensive line broadening and widespread loss of backbone signal intensity (Figure 6A,B, and Supplementary Figure S10B). This behavior is also consistent with a slow-to-intermediate exchange regime on the NMR timescale, although with more pronounced line broadening and signal attenuation than observed for TgPRP18p. Therefore, the interaction was primarily analyzed by monitoring residue-specific intensity losses as a function of peptide concentration. The most strongly affected regions largely coincide with those identified for TgPRP18p, indicating that both peptides engage a common interaction surface on TgCyp23 (Figure 6C). Residue-specific intensity attenuation was globally fitted using the same reversible 1:1 binding model, yielding an estimated *K*_d_ of 3.0 ± 1.0 μM (Figure 6D), in good agreement with the corresponding ITC-derived value.

## Discussion

TgCyp23 is a Cyp encoded by *T. gondii* which has been extensively characterized from a biochemical and structural perspective by our laboratories in recent years [20,23]. However, its precise biological function remains unknown.

Our data identify TgCyp23 as a strong candidate spliceophilin in *T. gondii* and provide evidence that this protein has retained the key molecular features previously described for the human spliceosomal hCypH. In the human system, hCypH forms stable complexes with PRP4/U4-U6-60K (a core component of the U4/U6-U5 tri-snRNP involved in early spliceosome assembly) and PRP18 (a splicing factor required for the second catalytic step of pre-mRNA splicing) through a binding surface that is distinct from the canonical Cyp catalytic pocket, enabling protein–partner recognition while preserving enzymatic activity [5].

The present work extends this paradigm to apicomplexan parasites, and specifically to *T. gondii*, where TgCyp23 emerges as a functional homolog of spliceosomal Cyps. The high sequence similarity between TgCyp23 and hCypH, together with conservation of the residues previously implicated in hPRP4/hPRP18 recognition, suggested a spliceosome-associated role for TgCyp23, and our biophysical and structural results now provide direct experimental support for this hypothesis.

The crystal structures of the TgCyp23–TgPRP4p complex and of the ternary TgCyp23–TgPRP4p–CsA complex show that the parasite protein recognizes TgPRP4p through the same overall face used by hCypH to engage its spliceosomal partners, while leaving the CsA-sensitive catalytic region fully accessible. This indicates that the bifunctional architecture of spliceosomal Cyps (partner binding on one surface and isomerase chemistry on another) is conserved across large evolutionary distance.

A particularly interesting aspect of the TgCyp23–TgPRP4p structure is the conservation of the recognition mode centered on the aromatic anchor residue of the peptide. In the human complex, the PRP4-derived peptide binds remotely from the active site, with insertion of a conserved phenylalanine side chain into a hydrophobic cavity on the Cyp surface, thereby defining a second protein-interaction site that is functionally separable from peptidyl-prolyl isomerase catalysis [7,24]. Our TgCyp23 structure reveals the same principle; Phe109 of TgPRP4p is buried in a hydrophobic cleft formed by TgCyp23 residues Pro89, Ile90, Gly91, Tyr92, and Lys93, and stabilized by a network of backbone and side-chain interactions that promote a folded bound conformation. At the same time, the subtle rearrangements observed around the TgCyp23 Lα2–β3 loop indicate that the parasite ortholog preserves a degree of local adaptability that may tune affinity and partner discrimination without requiring global structural reorganization.

Solution NMR experiments further corroborate these findings and extend them to TgPRP18. Both TgPRP4p and TgPRP18p perturb largely overlapping regions of TgCyp23, demonstrating that they engage a common interaction surface. Together with the crystal structures, these observations support a model in which TgCyp23 recognizes multiple spliceosomal partners through a conserved exosite while maintaining the ability to discriminate between them.

Both TgPRP4p and TgPRP18p bind TgCyp23 with low- to mid-micromolar affinity (*K*_d_ = 3.3 ± 0.3 μM and 18 ± 5 μM, respectively), consistent with specific but reversible binding. Notably, the affinity of TgPRP4p closely matches that reported for the human hCypH–PRP4 interaction (∼2 μM) [7]. In both cases, binding is strongly enthalpy-driven and opposed by an unfavorable entropic contribution, indicative of enthalpy–entropy compensation. This thermodynamic signature is characteristic of interactions involving folding-upon-binding of intrinsically disordered regions, in agreement with CD data showing that the peptides are largely disordered in solution but acquire α-helical structure upon binding. MD analyses further support this model by revealing stable complexes and highlighting distinct interaction patterns, including additional electrostatic contacts in the TgPRP4p complex involving its N-terminal Arg residue and residues near the TgCyp23 C-terminus and Glu82. While these differences do not translate into clearly distinguishable thermodynamic signatures, they point to subtle variations in the interaction network at the binding interface.

The difference in affinity between TgPRP4p and TgPRP18p may have functional implications within the spliceosome. Spliceosomal protein–protein interactions are typically transient and dynamically regulated, allowing the rapid assembly and disassembly of complexes [25,26]. In humans, PRP4 is involved in spliceosome assembly and activation during early stages, whereas PRP18 functions at later stages and is required for the second transesterification reaction of splicing. These distinct stage-specific roles may require different interaction dynamics. Consistent with this interpretation, PRP18 has been shown to interact with hCypH in biochemical studies [24], but has not been detected in complex with hCypH in isolated spliceosomal assemblies to date, suggesting that this interaction, although functionally relevant, is likely transient [27–29]. The tighter binding of TgPRP4p to TgCyp23 may therefore reflect a more stable interaction required during early spliceosome assembly, whereas the weaker TgPRP18p interaction may be better suited for transient engagement during later catalytic stages. Although this interpretation remains speculative in the absence of full-length proteins and spliceosome-level data, it aligns with the known stage-specific roles of PRP4 and PRP18 in the human system [30].

**Figure 7:**
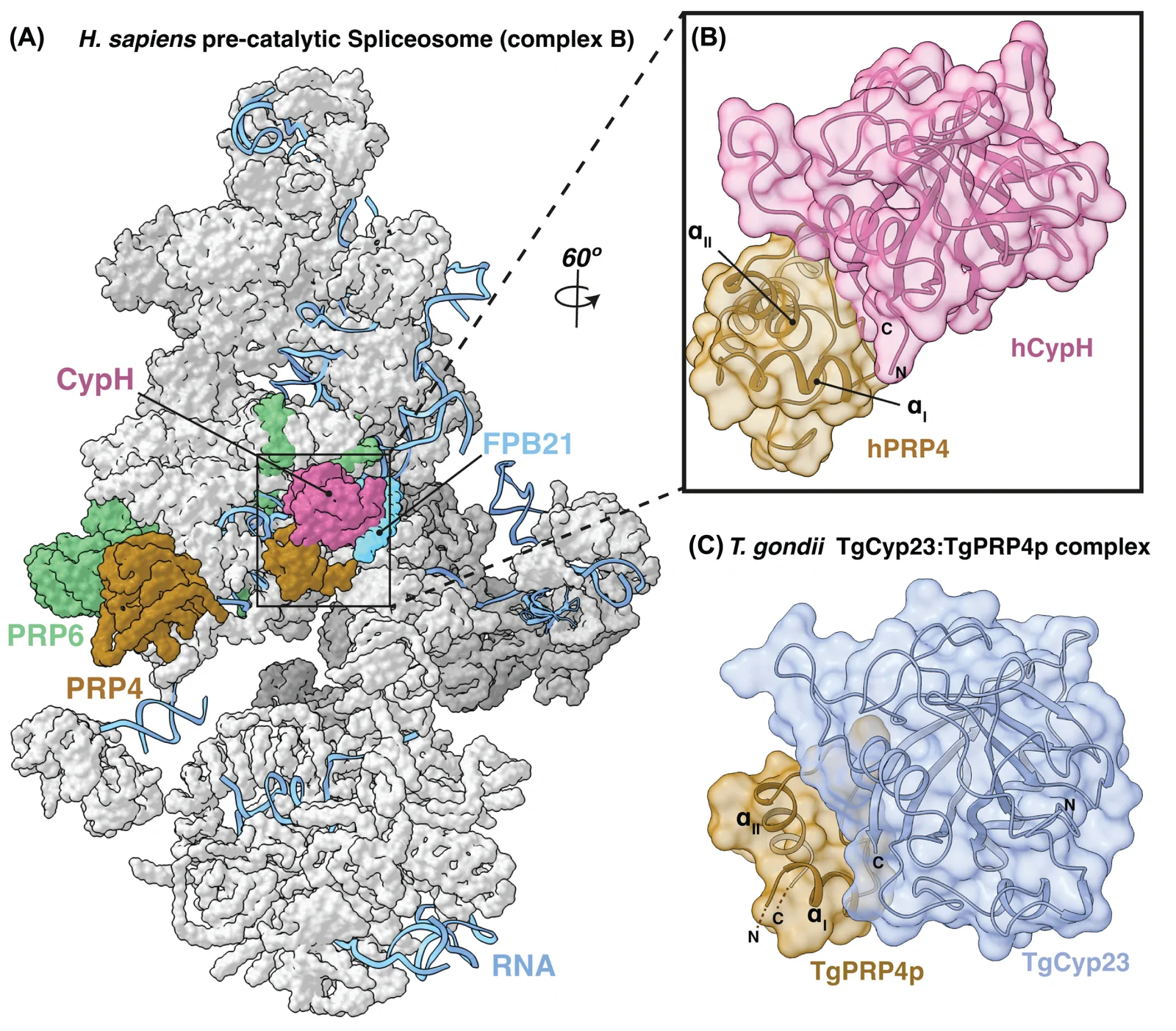
Spliceosome comparison between *H. sapiens* and *T. gondii* (**A**) Cryo-EM structure for human pre-catalytic complex B (PDB: 6AHD). Proteins are represented with a soft surface. hCypH is highlighted in pink, and partners PRP4, PRP6, and FPB21 are colored in orange, green, and cyan, respectively. Some RNA chains are observed through the structure, and are represented with blue cartoon. (**B**) Zoomed and 60° rotated-view of human CypH and PRP4 interaction. The complex is shown as cartoon, with a soft transparent surface. (**C**) Structure of the *T. gondii* TgCyp23–TgPRP4p complex (this work). The complex is represented in the same orientation as in panel (B). The structures are shown as cartoons with transparent surfaces. TgCyp23 is colored in blue, whereas TgPRP4 peptide is maintained in orange, as it is the structural homolog of hPRP4. α denotes α-helices, C and N indicate C- and N-terminal ends.

Importantly, binding of TgPRP4p or TgPRP18p does not affect TgCyp23 catalytic activity, and CsA remains a potent inhibitor even in the presence of bound peptide. This functional independence is explained by the structural separation between the PRP-binding surface and the catalytic pocket, consistent with the mechanism described for hCypH [5,24]. hCypH forms distinct complexes with hPRP4 and hPRP18, supporting its role as a modular interaction platform during spliceosome assembly and remodeling rather than merely as an isolated prolyl isomerase. Our data support a similar role for TgCyp23, which is likely to contribute to spliceosome organization by acting as a scaffold that stabilizes the association of specific spliceosomal components and facilitates their recruitment through a dedicated exosite while retaining catalytic competence for other proline-containing substrates (Figure 7).

The identification of a structurally distinct PRP-binding exosite is also relevant considering the growing appreciation that Cyps often use surfaces outside the canonical active site to achieve isoform-specific biology. Broad structural analysis of the human Cyp family has shown that regions adjacent to, but distinct from, the conserved catalytic pocket contribute substantially to molecular specificity and therefore represent promising opportunities for selective ligand design. More recently, subtype-selective Cyp inhibitors have been developed by exploiting exosite differences near the S2 region rather than relying solely on the highly conserved CsA-binding pocket [31,32]. In the case of TgCyp23, the discovery of a defined PRP-binding exosite opens the possibility that spliceosome-associated parasite Cyps might be targeted through interface-directed strategies, although the strong conservation with hCypH may limit parasite selectivity.

From a biological standpoint, our results place TgCyp23 within a pathway that is increasingly recognized as important for parasite fitness and pathogenicity. The *T. gondii* genome is intron-rich and relies on a conserved but evidently essential splicing apparatus. Recent work has shown that depletion of the core splicing factor Cdc5 causes major transcriptome disruption in *T. gondii*, underscoring the parasite dependence on robust pre-mRNA splicing [15]. Likewise, pharmacological targeting of TgPRP4K in *T. gondii* and PfCLK3 in *P. falciparum* with altiratinib causes widespread splicing defects and blocks parasite development, demonstrating that spliceosome-associated functions can be exploited therapeutically in apicomplexans. Additional support comes from the observation that the splicing factor SR2 contributes to *T. gondii* virulence *in vivo* [12,13,15]. Against this background, identifying TgCyp23 as a likely spliceophilin is not simply a matter of annotation; it expands the set of parasite splicing factors with experimentally defined molecular functions and strengthens the broader concept that the apicomplexan spliceosome contains biologically important components that could serve as therapeutic targets.

Although our data establish direct binding of TgCyp23 to PRP4- and PRP18-derived motifs *in vitro* and strongly support a spliceosomal role, several questions remain. The extent to which TgCyp23 is incorporated into native parasite spliceosomal particles, the precise stage of the *T. gondii* splicing cycle at which these interactions occur, and whether TgCyp23 follows a trajectory similar to that of hCypH or has evolved stage-specific adaptations in the parasite context remain to be clarified. In addition, while the use of short peptides captures the core recognition determinants, it may not fully reflect affinity, regulation, or post-translational control in the context of full-length TgPRP4 and TgPRP18.

In conclusion, the structural, kinetic and biophysical data converge on a model in which TgCyp23 is a spliceophilin-like Cyp that recognizes TgPRP4 and TgPRP18 through a conserved non-catalytic surface while preserving an accessible and inhibitor-sensitive active site. This organization closely parallels the architecture established for hCypH and supports the idea that core principles of spliceophilin-mediated recognition have been conserved in *T. gondii*. More broadly, these findings provide a molecular entry point for dissecting spliceosome-associated Cyp function in apicomplexan parasites and reinforce the emerging view that parasite RNA-processing machineries represent promising targets for mechanistically informed therapeutic intervention.

## Materials and methods

### Chemicals and materials

All standard chemical reagents were purchased from Sigma–Aldrich (St. Louis, MO, U.S.A.), Thermo Fisher Scientific (Waltham, MA, U.S.A.), and MedChemExpress (South Brunswick, NJ, U.S.A.), unless stated otherwise, and used as supplied. Synthetic peptides (Supplementary Table S1) were purchased from GenScript (Piscataway, NJ, U.S.A.).

### Recombinant protein production

TgCyp23 and TgCyp21 were expressed and purified as described previously [19,20,23]. TgCyp18 (Uniprot ID: S8F7V1; ToxoDB: TGME49_221210) was expressed following the protocol used for TgCyp23. Labeled ^15^N–TgCyp23 for NMR experiments was prepared in M9 minimal medium supplemented with ^15^NH_4_Cl (1 g l^–1^).

### Crystallization of the TgCyp23 complexes

For crystallization, the enzyme was buffer-exchanged into 50 mM Tris–HCl pH 7.5 containing 0.1 mM dithiothreitol (DTT). TgCyp23 was concentrated to 26 mg ml^−1^ using a Centricon-10 device. To obtain the binary TgCyp23–TgPRP4p complex, TgCyp23 was incubated with TgPRP4p at a final concentration of 1.5 mM, in a 1:1.3 molar ratio, respectively, and stirred for 24 h at 4°C. Crystallization assays were performed using the sitting-drop vapor diffusion method by mixing 0.2 μl of protein solution with 0.2 μl of precipitant solution. Crystals grew at 18°C after three days in a commercially available condition (Jena Bioscience) containing 0.1 M Bis–Tris pH 5.5, 100 mM ammonium acetate, and 17% (w/v) polyethylene glycol 10,000. To obtain the ternary TgCyp23-TgPRP4p–CsA complex, TgCyp23 was first incubated with solid CsA at a 1:8 molar ratio and stirred for three days at 4°C. The solution was then filtered through a 0.25 μm filter to remove the excess of solid CsA and subsequently incubated with TgPRP4p (1.5 mM) for 24 h at 4°C. Crystallization experiments were set up as described above by mixing 0.2 μl of protein solution with 0.2 μl of precipitant solution. Crystals appeared after three days in a commercial solution (Jena Bioscience) containing 0.1 M TBG buffer (Sodium Tartrate dihydrate, Bis–Tris, and Glycylglycine) pH 4.0 and 25% (w/v) polyethylene glycol 1,500. Finally, crystals were flash-frozen in liquid nitrogen.

### Data collection and structural determination of the protein–ligand complexes

Diffraction data sets were collected in the XALOC beamline at the ALBA synchrotron (Barcelona, Spain) using a Pilatus 6 M detector and a fixed wavelength of 0.97926 Å. Collected images were processed using autoPROC [33], XDS [34], and scaled using AIMLESS [35] from the CCP4 package [36,37]. TgCyp23–TgPRP4p crystals diffracted up to 2.4 Å resolution and belonged to tetragonal P 4_1_2_1_2 space group. There is one complex chain in the asymmetric unit with a solvent percentage of 52%. Structural determination was performed by molecular replacement method with Phaser MR of CCP4 [37], using the TgCyp23 structure (PDB: 8B58). Then, TgPRP4 peptide was manually placed using Coot by superimposing the AlphaFold model predicted for TgCyp23–TgPRP4p complex, which correctly anticipated the peptide-binding position observed in the crystallographic structure. On the other hand, crystals of the TgCyp23–TgPRP4p–CsA complex diffracted to 1.25 Å resolution and belonged to the triclinic space group P1, with a solvent content of 39% and two complex chains in the asymmetric unit. Structure determination was performed following the same procedure described above, using the TgCyp23–CsA structure as the search model in the molecular replacement. The high resolution of our crystallographic data allowed us to refine using anisotropic B-factors. Both structures were refined with REFMAC5 [38] and PHENIX [39,40] programs. All statistics for data processing and refinement are shown in the supplemental material in Supplementary Table S4.

### Isothermal titration calorimetry

Isothermal titration calorimetry (ITC) experiments were performed using a MicroCal PEAQ-ITC instrument (Malvern Panalytical Ltd., Malvern, UK) at 25°C [41,42]. Protein–peptide interactions were measured by titrating peptide solutions into the protein sample cell. Peptides were initially dissolved in deionized water at a final concentration of 5 mM, centrifuged at 10,000 rpm for 20 min before use, and diluted to 0.4 mM in ITC buffer (50 mM Hepes, pH 8.0, 100 mM NaCl). The pH of the final peptide solution was carefully verified to exclude any pH variation. A total of 25 μM protein in a volume of 200 μl was loaded into the sample cell and titrated with 19 injections of the 0.4 mM peptide solution (2 μl per injection). For experiments involving TgCyp23 pre-incubated with CsA, the protein was incubated with the inhibitor (dissolved in ethanol) at a 1:2 protein-to-inhibitor molar ratio prior to titration. To ensure solvent matching, an equivalent volume of ethanol was added to the peptide solution in the syringe. All experiments were conducted in 50 mM Hepes pH 8.0, 100 mM NaCl. All solutions were thoroughly degassed before use, and the pH was carefully verified to exclude pH variations during the measurements. Control experiments (injecting ligand solutions into the buffer) were conducted to obtain a baseline for each experiment and determine the heat of dilution/mixing. These values were subtracted from the experimental runs in the presence of protein. Data analysis was performed using the MicroCal PEAQ-ITC Analysis Software (Malvern Panalytical Ltd.) to obtain the apparent dissociation constant (*K*_d_), binding stoichiometry (N), enthalpy change (∆*H*), and entropy change (∆*S*). Reported values represent mean ± standard error of the mean (SEM) from at least three independent titrations, performed using at least two independent protein preparations.

### Circular dichroism spectroscopy

CD spectra were acquired on a Jasco J-1500 CD spectropolarimeter equipped with a Peltier-controlled thermostated cell holder, as previously described [43]. Far-UV CD spectra (190–260 nm) were acquired for TgCyp23 at a concentration of 0.2 g l^–1^ (8.7 μM) and for interactor peptides at 10.4 or 43.5 μM in deionized water using a quartz cuvette with a 0.1-cm path length. To assess conformational changes induced by peptide binding, TgCyp23 and peptides were incubated at a 1:1.2 and 1:5 protein-to-peptide molar ratio for 1 h at room temperature, and the spectra were recorded as described above.

CD spectra in the presence of increasing concentrations of 2,2,2-trifluoroethanol (TFE) were collected as described [44]. Peptide samples were prepared at 43.5 μM in deionized water and titrated with increasing amounts of TFE up to 80% (v/v), followed by equilibration for 5 min prior to spectral acquisition.

Thermal denaturation experiments were performed under the same conditions used for far-UV CD measurements. Temperature-dependent CD signals were monitored at 224 nm over a temperature range of 15–100°C, using a scan rate of 1.5°C min^−1^ and a response time of 4 s [45].

### PPIase assay

PPIase activity, both in the absence and in the presence of peptides, was measured using the synthetic substrate *N*-succinyl-Ala-Ala-Pro-Phe-*p*-nitroanilide (AAPF) in a chymotrypsin-coupled assay, as previously described [19,20,23,46]. All reactions were conducted at 0°C in 50 mM Hepes pH 8, 100 mM NaCl. The enzyme was used at a final concentration of 7 nM and mixed with 50 μM AAPF, solubilized in 470 mM LiCl and 100% TFE. To evaluate the effect of peptide binding on PPIase activity, TgCyp23 was pre-incubated at room temperature for at least 10 min with 25-, 50-, or 100-fold molar excess of each peptide prior to substrate addition. The total reaction volume was 200 μl, prepared in a 1-cm path cuvette. Once the reaction mixture reached 0°C, chymotrypsin dissolved in assay buffer was added at a final concentration of 1.8 g l^−1^ to initiate the reaction. The release of *p*-nitroaniline was monitored by measuring absorbance at 390 nm (ɛ_390 nm_ = 13300 M^−1^ cm^−1^) until completion of the reaction (∼1 min). Absorbance values were recorded every 0.2 s using a Jasco V-750 spectrophotometer (Tokyo, Japan). In all experiments, the reaction slope was corrected for spontaneous *cis*-to-*trans* isomerization in the presence of chymotrypsin alone.

### System preparation and molecular dynamics simulations

Molecular models of the TgPRP4, TgPRP18 and their respective complexes with TgCyp23 were created with the AlphaFold3 server [47]. Five initial molecular models for each species were created.

Modeled segments of each protein can be found in Supplementary Table S4.

Energy minimization and unrestrained MD simulations of the resulting all-atom models were run in explicit physiological saline solution under periodic boundary conditions using the *ff14SB* AMBER force field [48], a cutoff distance of 9 Å for the non-bonded interactions, the SHAKE algorithm for all bonds involving hydrogens and an integration step of 2.0 fs. Electrostatic interactions were represented using the smooth particle mesh Ewald method with a grid spacing of 1 Å. The simulation protocol made use of the *pmemd.cuda_SPFP* engine implemented in AMBER 18 [49] running on Nvidia GTX 1080 RTX2080Ti GPUs. Solvent molecules and counterions were first allowed to redistribute around the positionally restrained solute (5 kcal mol^−1^Å^−2^) during a heating phase (0.1 ns) from 100 to 300 K. Each system was then allowed to equilibrate at 300 K for 2.5 ns in the presence of weak restraints (0.5 kcal mol^−1^Å^−2^) on the protein Cα atoms and further simulated in the absence of any restraints for at least 300 ns. System coordinates were collected every 5 ns and subjected to a simulated annealing protocol (from 300 to 100 K over 1 ns) followed by energy minimization to improve convergence and reduce background noise [50,51].

### Analysis of the MD trajectories

Root-mean-square fluctuations per residue, as well as phi and psi torsion angles, were monitored with the aid of the *cpptraj* module in AmberTools [50], whereas estimations of the solvent-corrected peptide–protein binding energies, as well as their per-residue decompositions into van der Waals, coulombic, apolar, and desolvation contributions were provided by the MM-ISMSA software [52].

### NMR analysis of the interaction

Two-dimensional ^1^H–^15^N Heteronuclear Single Quantum Coherence (^1^H–^15^N HSQC) NMR spectroscopy experiments were acquired on a Bruker AVANCE NEO spectrometer operating at a proton Larmor frequency of 600 MHz and a temperature of 298 K, equipped with a triple-resonance Prodigy cryoprobe (Bruker BioSpin GmbH, Rheinstetten, DE). Uniformly ^15^N-labeled TgCyp23 was dissolved in 20 mM sodium phosphate buffer pH 7.4, 50 mM NaCl, containing 5% D_2_O. Peptides were dissolved directly in the same NMR buffer at a final concentration of 5 mM, centrifuged at 10,000 rpm for 20 min before use, and the pH was carefully verified to exclude any pH variation. Titration experiments were performed by gradually adding increasing amounts of each peptide to a 50 μM ^15^N–TgCyp23 sample up to a 3-fold peptide molar excess. After each titration step, ^1^H–^15^N HSQC spectra were acquired with a matrix of 2048 (^1^H) × 256 (^15^N) complex points. The CSP was calculated from the following equation:

$${\Delta \delta }_{HN}=\sqrt{\frac{{\left({\Delta \delta }_{H}\right)}^{2}+{\left({\Delta \delta }_{N}/5\right)}^{2}}{2}}$$

where *Δδ_H_* and *Δδ_N_* represent the observed chemical shift changes of the amide nuclei ^1^H and ^15^N upon ligand binding.

NMR data were processed using TopSpin v3.6.2 (Bruker) and NMRPipe v9.9, and analyzed with CcpNmr Analysis v2.5.2.

Binding affinities by NMR were estimated by monitoring the ligand-dependent attenuation of selected resonance intensities. Only resonances showing the smallest degree of exchange broadening were selected for the quantitative analysis. Residue-specific values of (1 − *I/I*_0_) were globally fitted using the following equation assuming a reversible 1:1 binding model. Data fitting was performed using GraphPad Prism 7.0c.

$$\left(1-\frac{I}{I_{0}}\right)=I_{\text{max}}\left[\frac{P_{0}+x+K_{d}-\sqrt{{\left(P_{0}+x+K_{d}\right)}^{2}-4\;P_{0}x}}{2P_{0}}\right]$$

## Supplementary Material

Supplementary Figures S1-S10 and Tables S1-S4

## Data Availability

The crystal structures and structure factors of the TgCyp23-TgPRP4p and TgCyp23-TgPRP4p-CsA complexes are available in the Protein Data Bank (PDB) under accession codes 29GR and 29GS, respectively [53, 54].

## Open Access

Open access for this article was enabled by the participation of Università degli Studi di Verona in an all-inclusive Read & Publish agreement with Portland Press and the Biochemical Society.

## Abbreviations

AAPF: *N*-succinyl-Ala-Ala-Pro-Phe-*p*-nitroanilide
Cyp: cyclophilin
CD: circular dichroism
CsA: cyclosporin A
hCypH: human cyclophilin H
ITC: isothermal titration calorimetry
MD: molecular dynamics
NMR: nuclear magnetic resonance
PPIase: peptidyl-prolyl cis–trans isomerase
SFM: splicing factor motif
TFE: 2,2,2-trifluoroethanol
