# Supplementary material for: A non-catalytic interaction surface mediates recognition of spliceosomal factors by the Toxoplasma gondii cyclophilin TgCyp23
Source: Biochem J. 2026 Sep 3;483(10):1799–816. doi: 10.1042/BCJ20260439 (PMC13543012; doi:10.1042/BCJ20260439)
Supplement: Supplementary file 1 [file BCJ-2026-0439_supp.pdf]

SUPPLEMENTARY INFORMATION

A non-catalytic interaction surface mediates recognition of spliceosomal factors by the *Toxoplasma gondii* cyclophilin TgCyp23

Filippo Favretto [a #], Silvia Fruncillo [a #], Eva Jiménez-Faraco [b, #], Federico Gago [c], Paola Dominici [a], Juan A. Hermoso [b, *], Alessandra Astegno [a, *]

[a] *Department of Biotechnology, University of Verona, Verona, Italy.*
[b] *Department of Crystallography and Structural Biology, Institute of Physical Chemistry Blas Cabrera (IQF), CSIC, Madrid, Spain.*
[c] *Department of Biomedical Sciences, School of Medicine and Health Sciences, University of Alcalá, Alcalá de Henares, Spain*

[#] These authors contributed equally.

*Corresponding authors. Alessandra Astegno, e-mail: alessandra.astegno@univr.it; Juan A. Hermoso, e-mail: xjuan@iqf.csic.es

TABLES

Table S1. Sequence alignment of TgPRP4p and TgPRP18p. Identical residues are marked with asterisks whereas amino acids involved in formation of an antiparallel β-sheet with TgCyp23 are connected by dots.

		Full protein ID
TgPRP4p	TVRKVLRALGHPICLFGEGPFERRQRLKVLF<br>`   * **    *::*:**    *  **  *`	Uniprot ID: S8F6Y9;<br>ToxoDB: TGME49_243540
TgPRP18p	EIFKKLRKMKEPVTLFGESDWQRYTRLCELE	Uniprot ID: A0A125YI37;<br>ToxoDB: TGME49_321290

Table S2. Apparent melting temperatures (Tm,app) of TgCyp23 and its complexes derived from CD thermal denaturation experiments.

**Sample**	**Tm,app**
TgCyp23	55 ± 1 °C
TgCyp23-TgPrp4	55 ± 1°C
TgCyp23-TgPrp18	54 ± 1 °C
TgCyp23-CsA	58 ± 1° C
TgCyp23-CsA-TgPrp4	57 ± 1°C
TgCyp23-CsA-TgPrp18	57 ± 1°C

Table S3. IC_{50} values for CsA inhibition of the PPIase activity of TgCyp23 and TgCyp23-peptide complexes.

**Complex**	**IC_{50} (nM)**
TgCyp23*	0.6 ± 0.1
TgCyp23- TgPRP18p	1.4 ± 0.8
TgCyp23- TgPRP4p	1.1 ± 0.5

*From F. Favretto, E. Jiménez-Faraco, C. Conter, P. Dominici, J. A. Hermoso, A. Astegno, "Structural Basis for Cyclosporin Isoform-Specific Inhibition of Cyclophilins from *Toxoplasma gondii*" *ACS Infect. Dis.* **2023**, *9*, 365–377.

Table S4. Data collection and refinement statistics for TgCyp23-TgPRP4p and TgCyp23-TgPRP4p-CsA complexes in this study.

	**TgCyp23-TgPRP4p**	**TgCyp23-TgPRP4-CsA**
**Data collection**	Isotropic	Anisotropic
Space group	$P4_12_12$	P1
Cell dimensions		
a, b, c (Å)	*a*=52.68, *b*=52.68, *c*=173.43	*a*=38.35, *b*=45.62, *c*=66.35
α, β, γ (°)	90, 90, 90	85.22, 83.94, 75.61
Wavelength (Å)	0.97926	0.97926
Resolution (Å)	45.06-2.40 (2.49-2.40)	44.15 - 1.25 (1.27 - 1.25)
unique reflections	10289 (1029)	396657 (19235)
R_{merge}	0.226 (2.007)	0.056 (2.027)
R_{pim}	0.054 (0.457)	0.035 (1.250)
$CC_{1/2}$	0.999 (0.824)	0.999 (0.524)
mean $I/\sigma(I)$	11.1 (1.8)	9.7 (0.7)
Completeness (%)	100.0 (99.9)	93.6 (90.1)
Multiplicity	19.0 (19.9)	3.5 (3.6)
**Refinement**		
Resolution (Å)	45.06-2.40	44.15-1.25
R_{work}/R_{free}	0.188/0.265	0.159/0.199
no. Of atoms		
Total	1829	4298
Protein	1803	3626
Ligands	-	205
solvent	26	467
**Ramachandran Analysis**		
Ramachandran favored (%)	96.92	98.01
Ramachandran outliers (%)	0.00	0.00
average B, all atoms ($Å^2$)	53.0	19.0
**Root-mean-square deviation (RMSD)**		
bond lengths (Å)	0.010	0.011
bond angles (°)	1.66	1.63
**PDB entry**	29GR	29GS

[a]Values between parentheses correspond to the highest resolution shells.

FIGURES

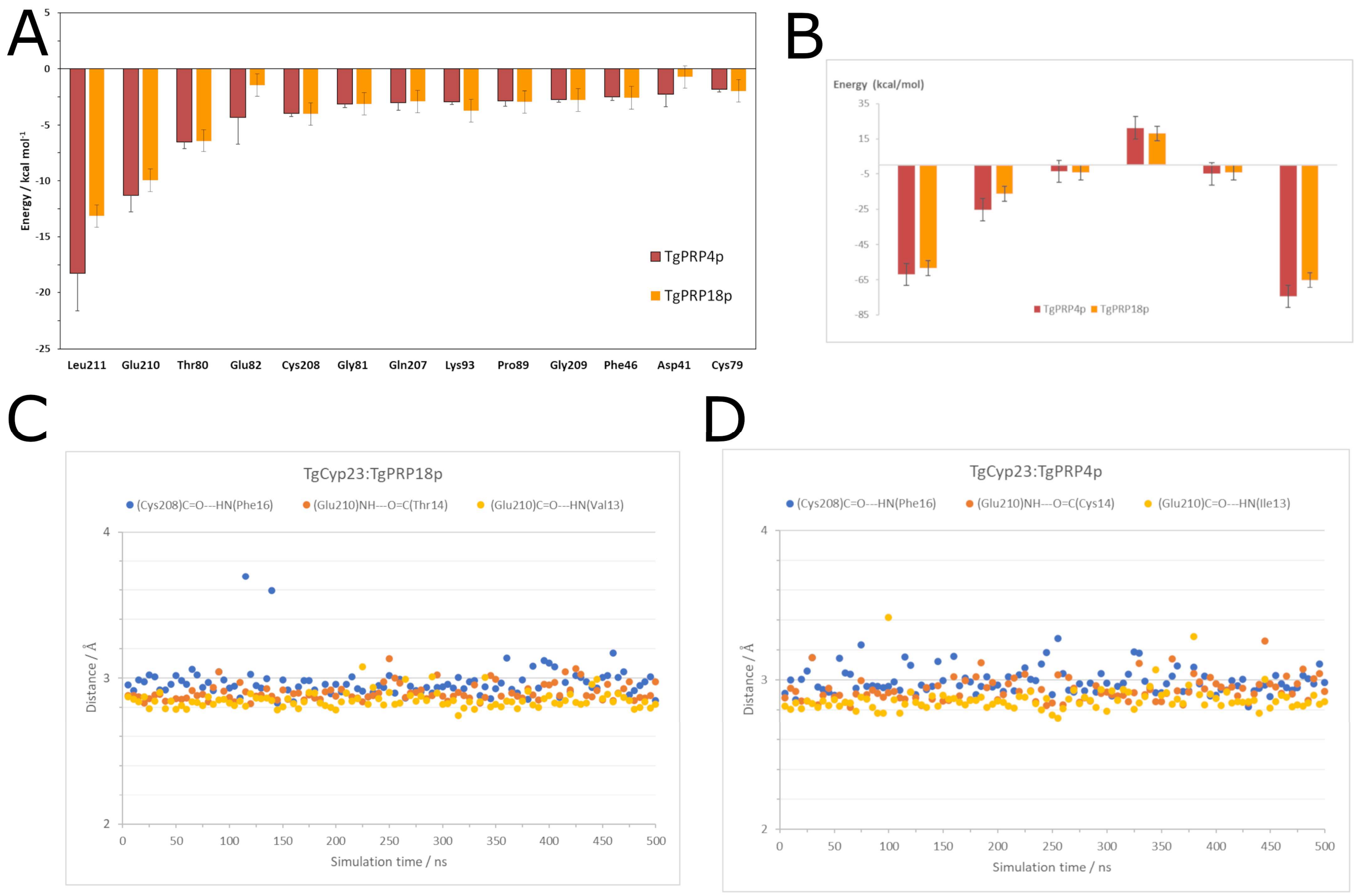

Figure S1. TgCyp23-TgPRP interaction energies. (A) Interaction energies (kcal mol^{-1}) between each TgPRP peptide studied and individual TgCyp23 residues. These values, which together represent a "binding fingerprint", were calculated by the MM-ISMSA program [Klett, J. et al., J Chem Theory Comput 2012]. A cutoff of 1.8 kcal mol^{-1} was used for improved clarity and display purposes. (**B**) Component contributions to the overall interaction energies between each TgPRP peptide studied and TgCyp23, as calculated by the MM-ISMSA program [Klett, J. et al., J Chem Theory Comput 2012]. The paired vertical blocks in the histogram correspond, from left to right, to van der Waals, electrostatic, peptide desolvation, TgCyp23 desolvation, apolar and total energy values. In both panels, the reported mean values and standard deviations were calculated from the 100 fully equilibrated, energy-minimized frozen complexes, which are expected to be closer to the global minimum structures. (**C**) Hydrogen bond distance variation for the three hydrogen bonds during MD simulations of the TgCyp23-TgPRP4p complex. Blue dots indicate the distance variation for Cys208(CO)–Phe16(HN) (corresponding to Phe109 in full-length TgPRP4); orange dots indicate Glu210(HN)–Cys14(CO) (corresponding to Cys107 in full-length TgPRP4); yellow dots indicate Glu210(CO)–Ile13(HN) (corresponding to Ile106 in full-length TgPRP4). (**D**) Hydrogen bond distance variation for the three hydrogen bonds during MD simulations of the TgCyp23-TgPRP18p complex. Blue dots indicate the distance variation for Cys208(CO)–Phe16(HN) (corresponding to Phe119 in full-length TgPRP18); orange dots indicate Glu210(HN)–Thr14(CO) (corresponding to Thr117 in full-length TgPRP18); yellow dots indicate Glu210(CO)–Val13(HN) (corresponding to Val116 in full-length TgPRP18).

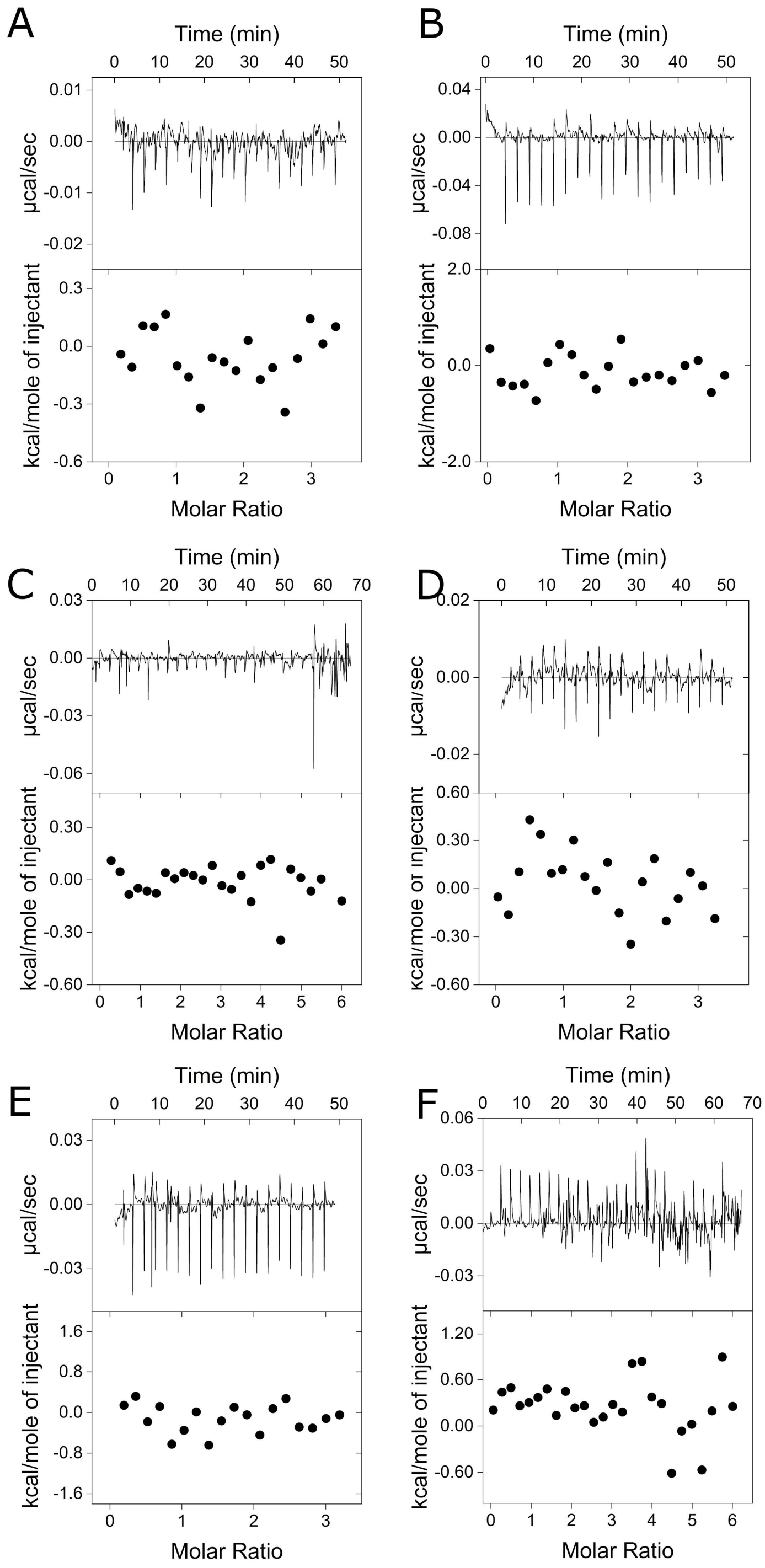

Figure S2. Control ITC experiments. (A) Representative control ITC experiment in which the sample cell contained TgCyp23 in the presence of 0.2% (v/v) ethanol and the syringe contained the corresponding buffer supplemented with the same ethanol concentration. (B) Representative control ITC experiment in which the sample cell contained TgCyp23 pre-incubated with CsA in the presence of 0.2% (v/v) ethanol and the syringe contained the corresponding buffer supplemented with 0.2% (v/v) ethanol. (C, D) Representative ITC experiments showing titration of TgPRP4p and TgPRP18p, respectively, into TgCyp18. (E, F) Representative ITC experiments showing titration of TgPRP4p and TgPRP18p, respectively, into TgCyp21. The negligible heat changes observed in panels A and B demonstrate that ethanol does not contribute significantly to the ITC signal under the experimental conditions, whereas panels C-F confirm the absence of detectable binding between the peptides and TgCyp18 or TgCyp21.

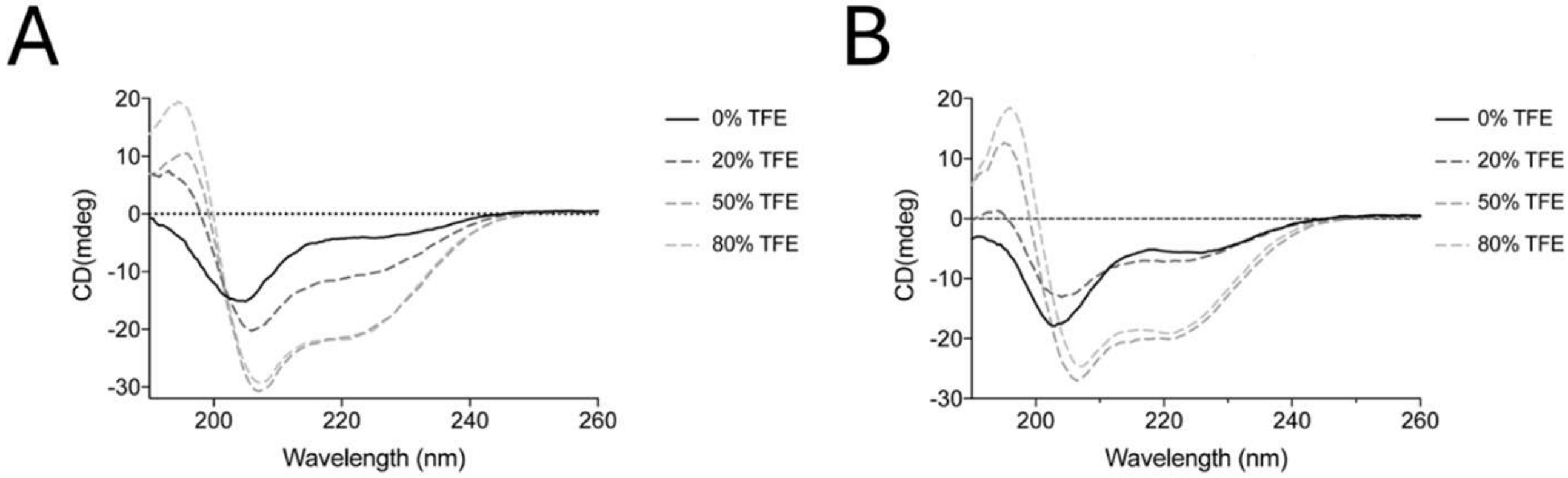

Figure S3. Intrinsic folding propensity of (A) TgPRP18p and (B) TgPRP4p. Peptide samples (43.5 μM) were titrated with increasing concentrations of TFE up to 80% (v/v) and equilibrated for 5 min prior to spectral acquisition.

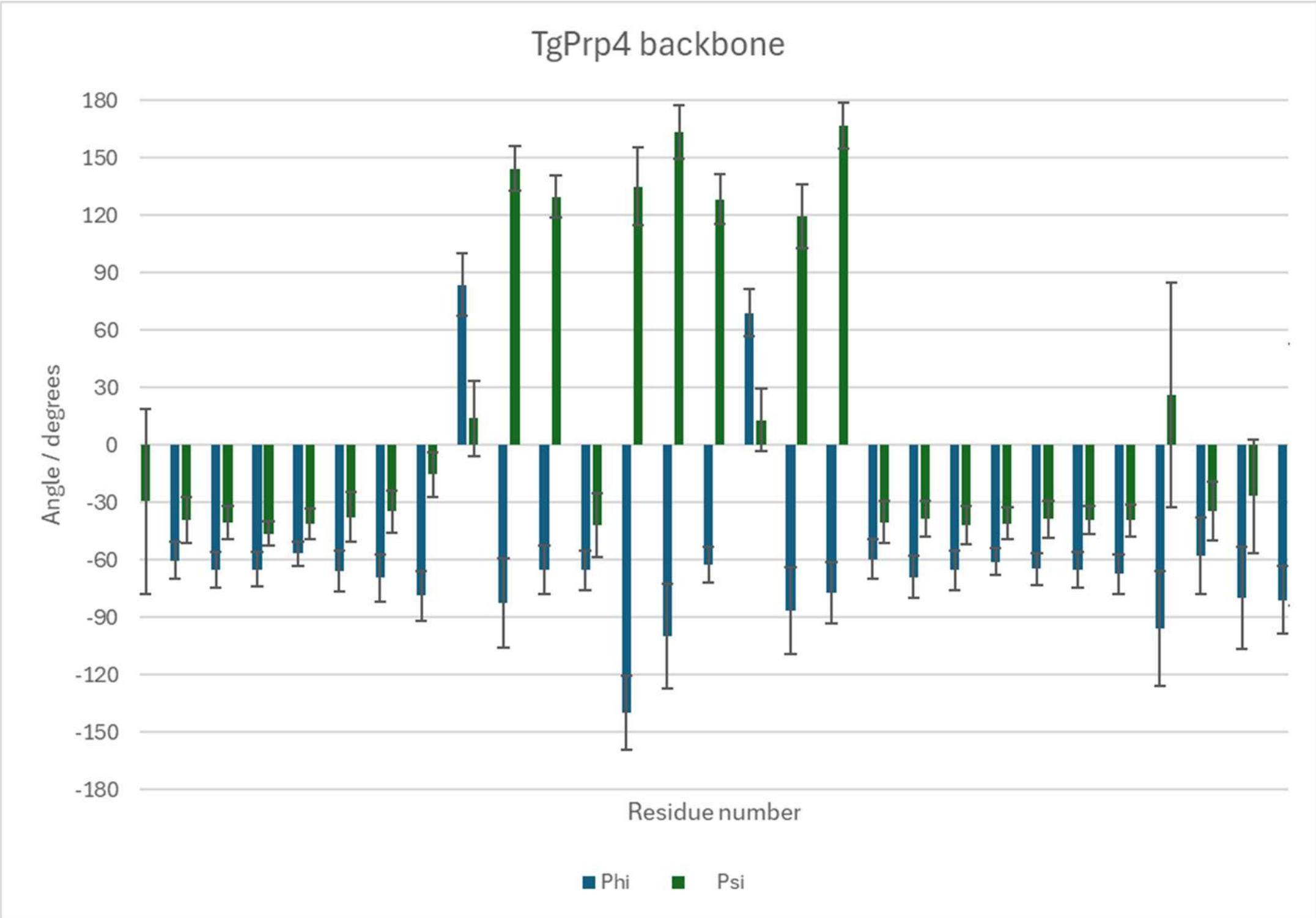

Figure S4. Average values (± s.d.) of the ϕ (phi) and ψ (psi) torsion angles of TgPRP4p that define the backbone conformation by representing the rotations around the N-Cα and Cα-C bonds, respectively.

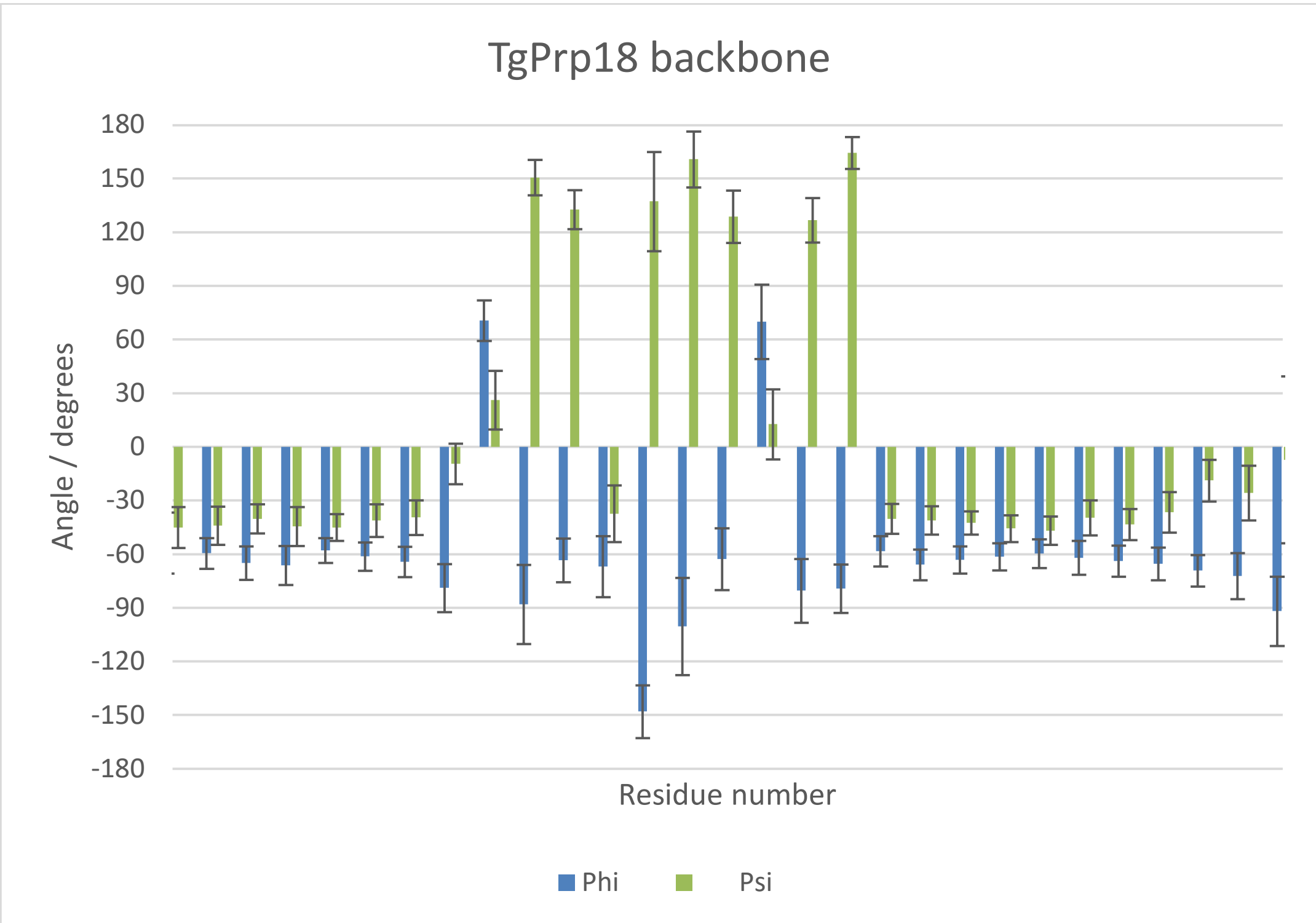

Figure S5. Average values (± s.d.) of the ϕ (phi) and ψ (psi) torsion angles of TgPRP18p that define the backbone conformation by representing the rotations around the N-Cα and Cα-C bonds, respectively.

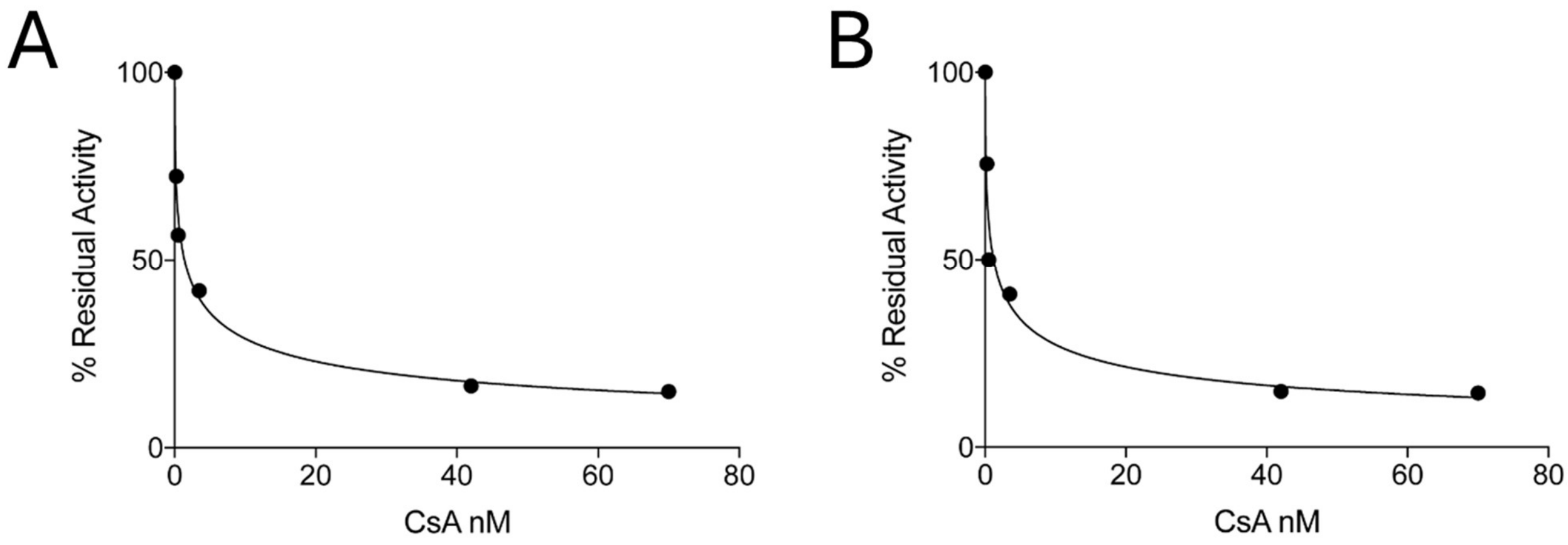

Figure S6. Representative PPIase inhibition curves for TgCyp23-peptide complexes in the presence of cyclosporin A (CsA). (A) TgCyp23-TgPRP18p complex; **(B)** TgCyp23-TgPRP4p complex. TgCyp23 was preincubated with a 50-fold molar excess of each peptide prior to addition of increasing concentrations of CsA.

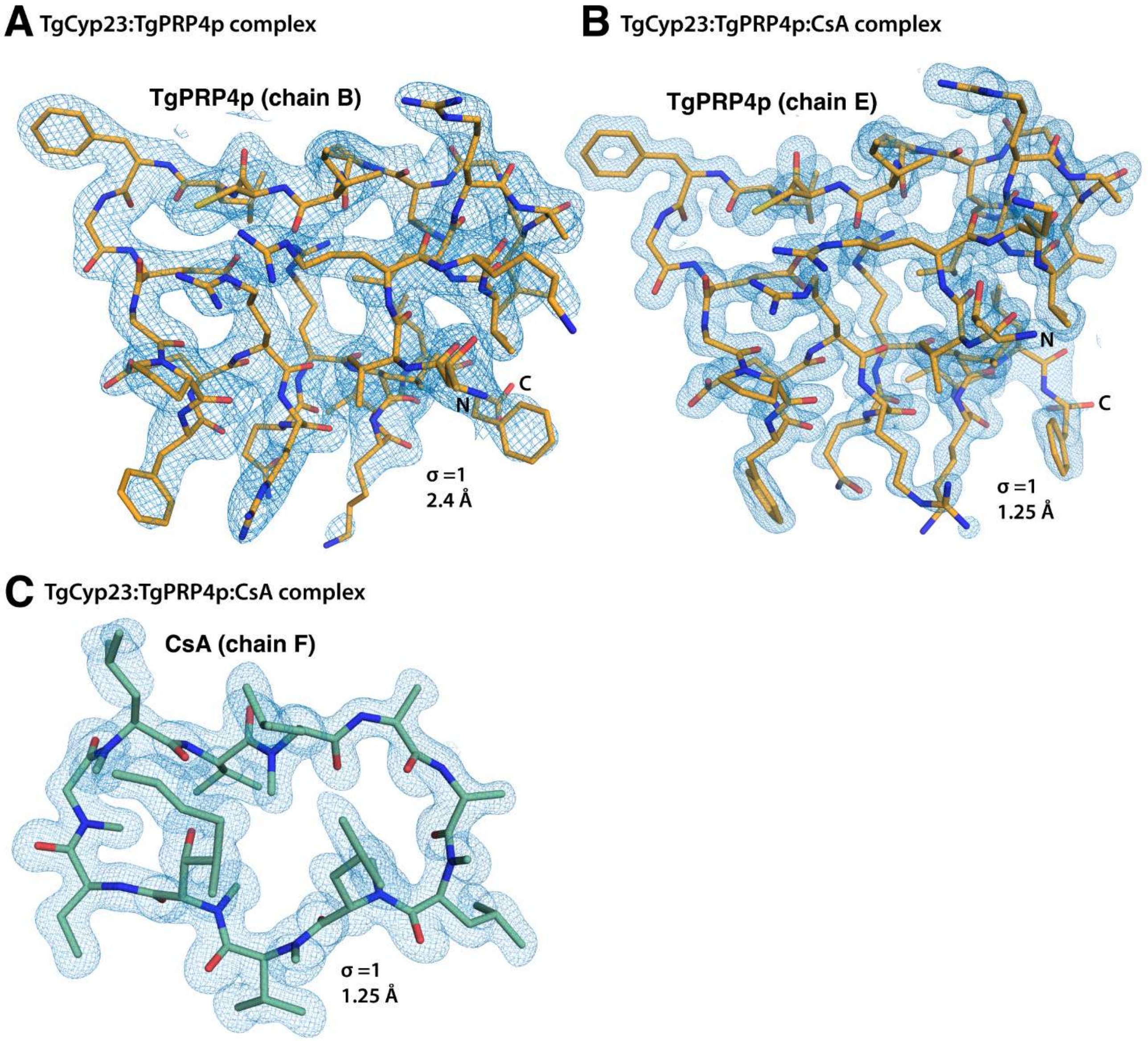

Figure S7. **2Fo-Fc Electron-density maps contoured at 1σ for the ligands in TgCyp23 complexes.** All molecules are shown as capped sticks. (**A**) Density map for TgPRP4 peptide in TgCyp23-TgPRP4p complex. (**B**) and (**C**) show the density maps for TgPRP4 peptide and CsA respectively, in the TgCyp23-TgPRP4p-CsA complex. The asymmetric unit of the TgCyp23-TgPRP4p-CsA crystal have two independent complex chains with the ligands clearly visible in both. We show the electron-density maps only for one chain as a representative example.

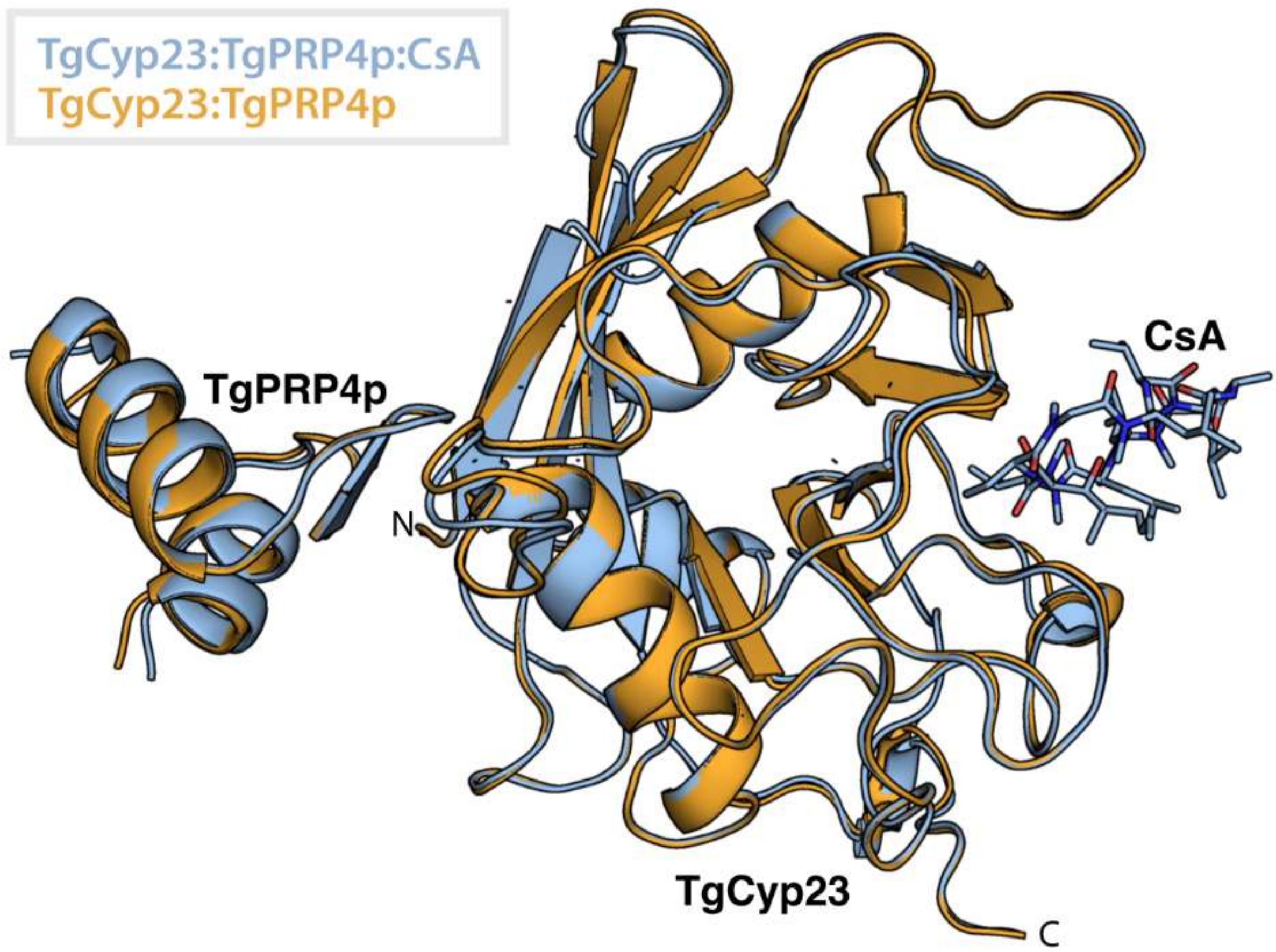

Figure S8. Alignment for the binary and ternary complexes of this study. TgCyp23-TgPRP4p and TgCyp23-TgPRP4p-CsA complexes are shown as orange and blue cartoons respectively. For the ternary complex we have selected one of the two TgCyp23 chains in the asymmetric unit (chain D). N and C denote the N- and C-termini, respectively.

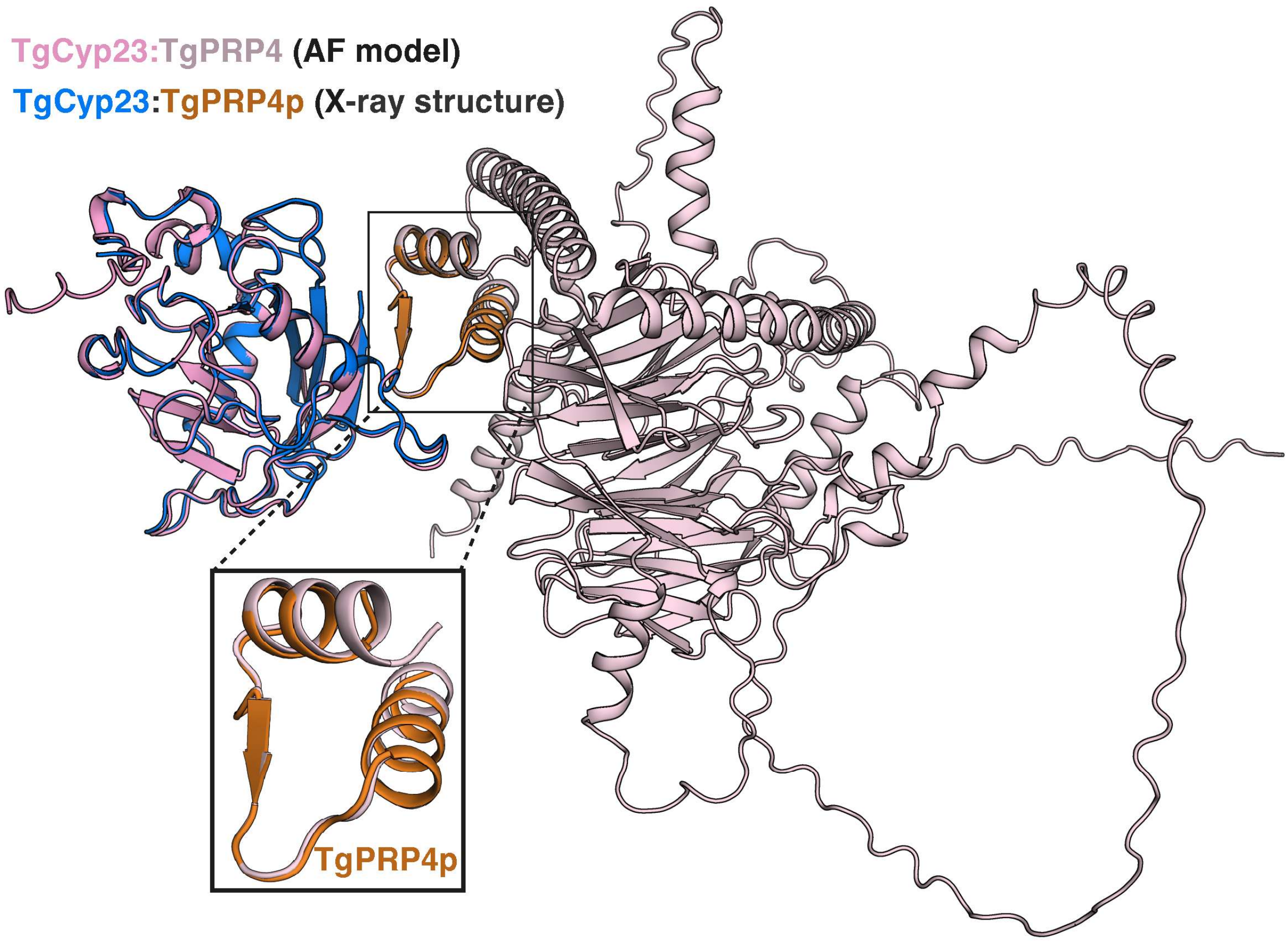

Figure S9. Structural alignment between crystallographic TgCyp23-TgPRP4p complex and AlphaFold prediction for TgCyp23-TgPRP4 full-length. AlphaFold model for the full-length prediction is colored in pink; the N-terminal region of TgPRP4 was predicted highly disordered and it has been omitted for clarity in the figure. TgCyp23-TgPRP4p crystallographic complex is colored in blue (TgCyp23) and orange (TgPRP4 peptide). In all five structural models generated by AF, the TgPRP4 region that is predicted to interact with TgCyp23 was located in the same position and retained the same fold. A zoomed-view of this region is represented in the black box.

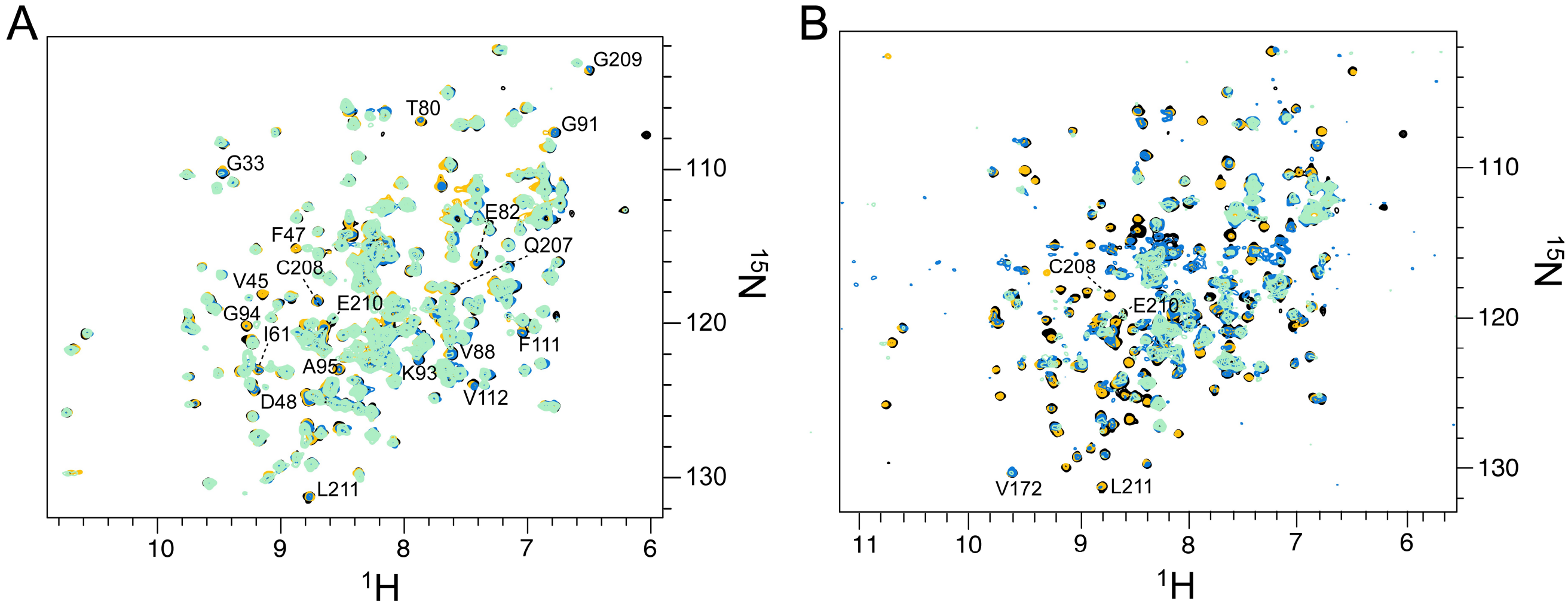

Figure S10. NMR spectroscopy analysis of the interaction between ^{15}N-TgCyp23 and TgPRP18p or TgPRP4p. (A) Superposition of ^{1}H-^{15}N HSQC spectra of ^{15}N-TgCyp23 recorded during titration with TgPRP18p at protein-to-peptide molar ratios of 1:0 (black), 1:0.5 (orange), 1:1.5 (blue), and 1:3 (light green). **(B)** Superposition of ^{1}H-^{15}N HSQC spectra of ^{15}N-TgCyp23 recorded during titration with TgPRP4p at protein-to-peptide molar ratios of 1:0 (black), 1:0.2 (orange), 1:1.5 (blue), and 1:3 (light green).
